# Key indicators of repetitive overuse-induced neuromuscular inflammation and fibrosis are prevented by manual therapy in a rat model

**DOI:** 10.1186/s12891-021-04270-0

**Published:** 2021-05-05

**Authors:** Mary F. Barbe, Michele Y. Harris, Geneva E. Cruz, Mamta Amin, Nathan M. Billett, Jocelynne T. Dorotan, Emily P. Day, Seung Y. Kim, Geoffrey M. Bove

**Affiliations:** 1 Department of Anatomy and Cell Biology, Lewis Katz School of Medicine, Temple University, 3500 North Broad Street, Philadelphia, PA 19140 USA; 2 Bove Consulting, Kennebunkport, ME 04046 USA

**Keywords:** Overuse injury, Work-related musculoskeletal disorders, Nerve, Muscle, Tendon, Massage therapy

## Abstract

**Background:**

We examined the effectiveness of a manual therapy consisting of forearm skin rolling, muscle mobilization, and upper extremity traction as a preventive treatment for rats performing an intensive lever-pulling task. We hypothesized that this treatment would reduce task-induced neuromuscular and tendon inflammation, fibrosis, and sensorimotor declines.

**Methods:**

Sprague-Dawley rats performed a reaching and lever pulling task for a food reward, 2 h/day, 3 days/week, for 12 weeks, while simultaneously receiving the manual therapy treatment 3 times per week for 12 weeks to either the task-involved upper extremities (TASK-Tx), or the lower extremities as an active control group (TASK-Ac). Results were compared to similarly treated control rats (C-Tx and C-Ac).

**Results:**

Median nerves and forearm flexor muscles and tendons of TASK-Ac rats showed higher numbers of inflammatory CD68+ and fibrogenic CD206+ macrophages, particularly in epineurium, endomysium and epitendons than TASK-Tx rats. CD68+ and CD206+ macrophages numbers in TASK-Tx rats were comparable to the non-task control groups. TASK-Ac rats had more extraneural fibrosis in median nerves, pro-collagen type I levels and immunoexpression in flexor digitorum muscles, and fibrogenic changes in flexor digitorum epitendons, than TASK-Tx rats (which showed comparable responses as control groups). TASK-Ac rats showed cold temperature, lower reflexive grip strength, and task avoidance, responses not seen in TASK-Tx rats (which showed comparable responses as the control groups).

**Conclusions:**

Manual therapy of forelimbs involved in performing the reaching and grasping task prevented the development of inflammatory and fibrogenic changes in forearm nerves, muscle, and tendons, and sensorimotor declines.

**Supplementary Information:**

The online version contains supplementary material available at 10.1186/s12891-021-04270-0.

## Introduction

Musculoskeletal disorders secondary to occupational overuse are highly prevalent in many professions [1–3]. These injuries and disorders have profound effects on the neuromuscular system (which includes nerves, muscles, and tendons) [4]. Acute tissue injury results in acute inflammatory cell infiltration, while repeated injury is associated with chronic inflammation and fibrosis in tissues undergoing chronic injury/repair processes [5, 6].

We have a rat model of occupational overuse injury, in which rats voluntarily reach and pull on a lever bar at target reach rates and force levels for a food reward [7]. In this model, operant reach rates and force levels were determined from studies on risk exposure to humans involved in occupational jobs [6, 8]. Prolonged performance of a high repetition high force lever-pulling task induces sensorimotor declines and neuromuscular inflammation (increased numbers of pro-inflammatory macrophages and cytokines) and fibrosis [7, 9–15], yet effective preventive treatments that can be provided long-term are still needed (i.e., concurrent with the continued “work”). Previously, we found that 8 weeks of ibuprofen treatment, concurrent with continued work, was helpful in the initial prevention of discomfort and tissue pathologies [9, 16, 17]. However, it was not successful long-term with 8 weeks of ibuprofen treatment (provided in task weeks 4–12) leading to drastically reduced task performance function and only partially rescued grip strength by week 12 [9, 16]. Additionally, the ibuprofen treatment and an anti-tumor necrosis factor drug failed to rescue task-induced somatosensory hypersensitivity [18, 19]. Prolonged use of anti-inflammatory drugs can lead to other problems, such as gastrointestinal or systemic toxicity in the case of ibuprofen [20] and immune suppression in the case of anti-tumor necrosis factor drugs [21]. In contrast, a manual therapy of the upper extremity (involving forearm skin rolling, gentle mobilization, upper extremity traction, deeper massage, and wrist joint mobilization) prevented task-induced tissue fibrosis and maintained grip strength in a long-term study [13], and nerve inflammation, fibrosis, and somatosensory hypersensitivity in a short-term study [12]. However, we have yet to determine a manual therapy treatment can prevent or reduce inflammatory responses in musculotendinous tissues, or somatosensory hypersensitivity, that develop with long-term performance of repetitive overuse tasks [7, 9, 10].

Others have examined the effects of various types of massage or manual therapy on inflammation in rodents and rabbits. Cyclical compression of rat and rabbit hindlimb has been shown to attenuate the number of cells expressing pro-inflammatory cytokines in muscles after prolonged hindlimb immobilization [22], and to reduce neutrophil and inflammatory macrophage (RAM-II+) infiltration into muscles exposed to intensive eccentric exercise [23–26]. In mice, active stretching has been shown to reduce neutrophil and inflammatory macrophage (CD68+) numbers in subcutaneous connective tissues after carrageenan injection [27, 28].

It has been suggested that the type of massage or manipulation used may affect the response in the involved tissue(s) [29]. Therefore, one of our goals in this study was to estimate the forces delivered by the subcomponents of treatments and to determine if the presumed less-intense components of our previous manual therapy protocol (specifically, forearm skin rolling, forearm muscle mobilization, and upper extremity traction) were able to prevent the development of tissue fibrosis and grip strength declines as effectively as our past combined treatment of both less- and more-intense components [12, 13]. We also extended our past 12 week study to now determine if this combination of manual therapy treatments could: 1) prevent or reduce inflammatory M1 type macrophage (CD68+) numbers in nerves, muscles and tendons; 2) increase IL-10 levels; 3) improve sensory hypersensitivity, since we did not examine such changes in our past 12 week manual therapy treatment study [13]; and 4) examine for the first time, the effects of a massage/manual therapy treatment on fibrogenic M2a type macrophages (specifically, CD206/mannose receptor immunopositive macrophages known to have fibrogenic roles [30–32].

The possibility that a relatively simple, widely available, and inexpensive prophylactic treatment could prevent the human disorders that this model replicates offers considerable potential to limit suffering, increase work product, and decrease lost work and disability costs associated with overuse disorders.

## Materials and methods

### Animals

Experiments were approved by the Temple University Institutional Animal Care and Use Committee in compliance with NIH guidelines for the humane care and use of laboratory animals. This study was also carried out in compliance with Arrive Guidelines [33]. Studies were conducted on 30 young adult (3 months of age at onset), female, Sprague-Dawley rats (Charles Rivers, Wilmington, MA, United States). Female rats were used to allow comparison to our past studies on female rats using this same model and related treatment [12, 13, 34].

Rats were food restricted to weigh 5% less than age-matched, normal control rats with free access to food (these latter rats were used for weight comparison purposes only). This food restriction was necessary to motivate the rats to work for a food reward. Thereafter, all rats were carefully maintained at the 5% food restriction for the duration of the experiment. Food restricted control (C) rats received similar amounts of rat chow and food reward pellets as TASK rats. All rats were weighed twice per week, provided regular rat chow daily in addition to food reward pellets (banana (F0024) and chocolate grain-based (F0165) dustless precision pellets, Bio-Serv, Flemington, NJ, United States), and allowed to gain weight over the course of the experiment (Supplemental Figure 1A, B). Animals received numbers, as names, at this time to conceal group allocation.

Twenty rats were operatively shaped to perform a high repetition high force task (TASK) and then randomly separated into two groups of 10 rats each. One group received treatment to both upper extremities (UE; TASK-Tx, *n* = 10) since rats use both limbs to perform this task [11, 12, 35]. The other group received a similar treatment to the lower extremities (LE), thus serving as an active control treatment (Task-Ac, *n* = 10). Ten control (C) rats that did not perform the task were separated into groups of 5, and received treatment to the UE (C-Tx, *n* = 5) or LE (C-Ac, *n* = 5).

### Repetitive task

Sixteen custom-designed operant behavioral chambers were used in which rats performed an operant reaching and lever pulling task, as previously described [19]. Briefly, in this study, task rats were operantly shaped for 5–6 weeks to learn the reaching and lever-pulling task at high force loads (ramping upwards from naïve, 10 min/day, 5 days/wk). Rats then performed a high repetition and high force reaching and lever-pulling task for a food reward at a target grasp force of 47% ± 5% (mean ± SEM) of their maximum pulling force (which is 1.44 ± 0.07 Newtons), at a target reach rate of 4 reaches/min, for 2 h/day, in 30 min intervals (with 1.5 h break between sessions), for 3 days/wk., for 12 weeks.

### Manual therapy

The manual therapy treatment protocol used was a subset of a previously developed protocol and included gentle forearm tissue mobilization, forearm skin rolling, and a gentle traction (stretch and glide) to the entire upper extremity (UE). The individual providing these treatments was the same as in previously published studies (MYH) [12, 13]. The protocol, designed to emulate what a massage therapist or other manual therapist might perform, consisted of scaled-down version of three commonly used modalities, as described next. We added a similar treatment to the lower extremity (LE) of TASK-Ac and C-Ac rats, as an active control for possible indirect effects of the manual therapy treatment. Treatments were performed 3 times per week, on alternate days to the task performance.

**Fig. 1:**
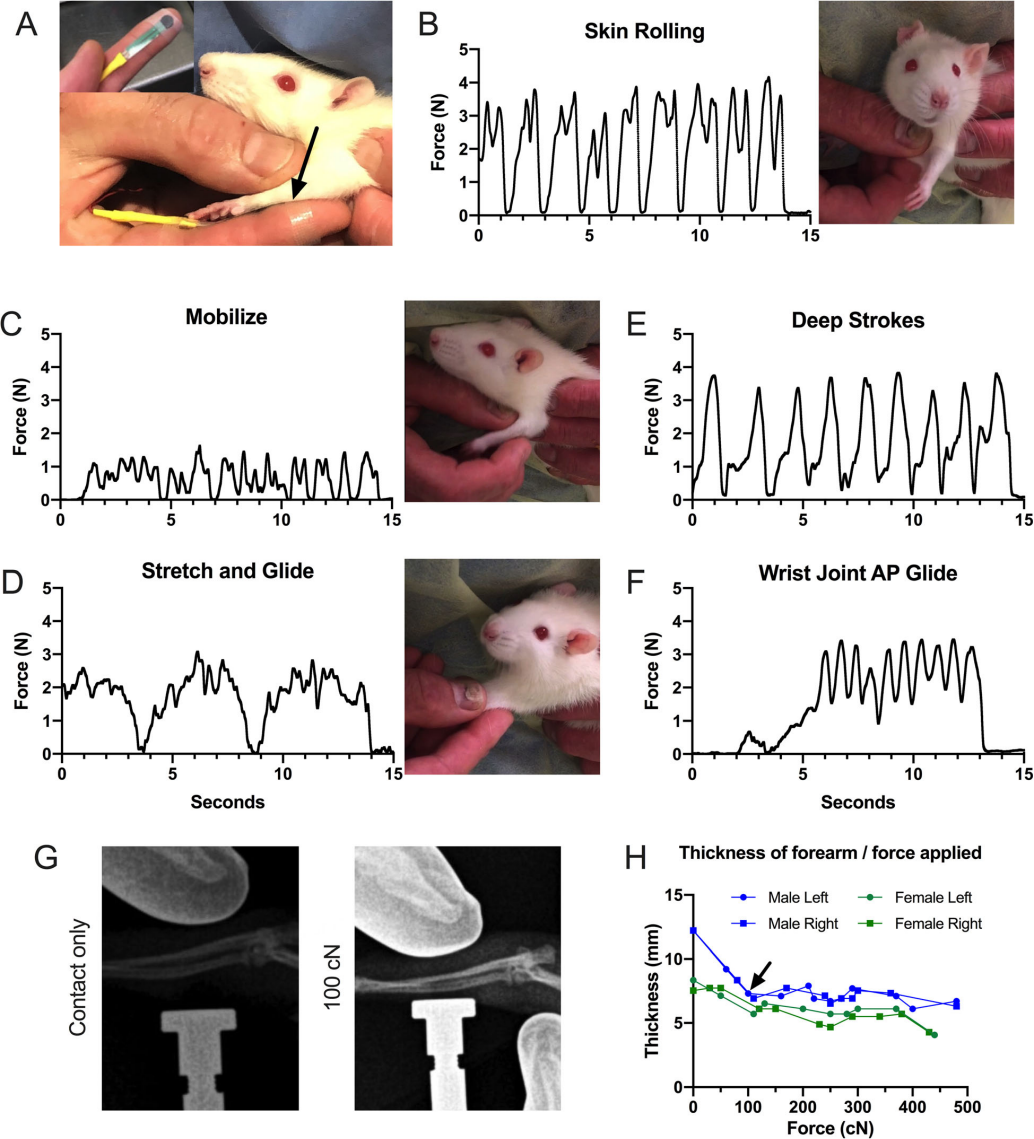
Forces delivered by treatments to forearm. **a** A flexible 5 mm diameter pressure sensitive resistor was taped to the tip of the treating index finger (as applied with inset showing detail). Each treatment component was performed, and forces recorded in Newtons (N). Arrow points to the placement of the flexible resistor in use. Caveats of this method include variable contact area and angle of application of the finger to the resistor and of the resistor to the rat while performing treatments. **b**, **c**, and **d** depict skin rolling, muscle mobilization, and upper extremity traction (stretch and glide), respectively. **e** and **f** show forces used in two components of the treatment used previously [13]. These latter two treatments were not included in the current protocol as an effort to test what we thought were “light” or “superficial” treatments, compared to “deep” treatments. However, as can be seen in these recordings, the “stretch and glide” treatment uses forces that are perpendicular to the forearm and in the general range of the “deep strokes” treatment. **g** Radiographs of the method used to measure forces when delivered during the “deep strokes” treatment, taken at light contact and during 100 cN force (see arrow in **h**). Bones shown are the radius and ulnar of the forearm. **h** Change of thickness during different forces applied to the forelimb

Our now-extensive experience with this treatment approach suggested that the treatment components that are considered superficial in humans and which we used as such in rats, were compressing and mobilizing the much smaller and mobile structures similarly as the seemingly more invasive past treatments, which had also included deep massage and wrist joint mobilization [12, 13]. We devised a method to estimate the forces delivered and help ensure consistency of the treatments. The output of a 5 mm diameter pressure-sensitive resistor (Trossen Robotics, Illinois, United States) was led to a bridge amplifier, digitized, sampled at 500 Hz, and recorded using Spike 2 software (Cambridge Electronic Designs, Cambridge, United Kingdom). The resistor was calibrated using a laboratory scale padded with a material of similar density to rat muscle. The resistor was placed between the finger of the operator and the skin of the rat, giving an estimation of the applied forces (Fig. 1a).

Manual therapy treatments were fashioned after common treatments used by massage therapists. Non-sedated rats were placed in the lap of the seated operator (MYH) and held gently until comfortable (1–2 min). The scapula and shoulder (for the UE treatments) or hip (for the LE treatments) were then stabilized with one hand while the other hand delivered the treatment in 3 phases:

1. Forearm Skin Rolling: The skin over the forearm region between the elbow and the wrist, or hindlimb region between the knee and the ankle, was pinched into a fold and rolled between the thumb and index finger (5 cycles per session, Fig. 1b), including all loose skin from the wrist to the elbow, or knee to ankle (for UE versus LE treatments).
2. Forearm Flexor Muscle Mobilization: The thumb, index finger, and middle finger gently compressed the UE forearm flexor muscles (Fig. 1c) or the LE gastrocnemius muscles, and the tissues were mobilized from side to side over the underlying bones in a rhythmic manner (10 cycles/treatment session).
3. Limb traction (stretch and glide): This treatment could only be performed when the rat relaxed their upper or lower extremities, which they did readily. While the torso was stabilized, the treating thumb and fingers gently grasped and compressed the rat’s proximal arm or thigh. The operator then gently tractioned the limb while simultaneously sliding their grasp down the rat’s upper limb, being careful not to pull the fur. Starting at the elbow or knee, the structures were rhythmically oscillated while continuing to the rat’s digits, until the digits slid out of the treating hand (5 tractions/treatment session, Fig. 1d). There were 10 cycles/treatment session. This treatment tractions the shoulder, elbow, wrist, and digit joints, passively stretches all forearm flexor muscles, and compresses the muscles of the forearm, or, tractions the hip, knee, ankle, and digits, and compresses the muscles of the gastrocnemius region of the hindlimb.

Treatments were performed bilaterally on either the UE and LE, and took less than 10 min per session including acclimation to the treatment provider, depending on the cooperation of the individual rat.

We also used x-ray imaging to determine the compression of the forearm soft tissues using forces similar to those recorded using the resistor (Fig. 1a-f). For the x-rays, anesthetized rats were positioned for lateral imaging, with the posterior forearm against the tip of a precision force gauge (Chatillon DFE II, Ametek, Florida, United States; Fig. 1g). The operator (for these experiments, GMB) wore radio-opaque gloves, and applied forces to the soft tissues of the forelimb that were similar to those delivered during the treatments (Fig. 1a and e). The distances at contact (no force) and at forces to 500 cN were measured, and plotted against the forces.

### Examination of indices of potential discomfort during the manual therapy treatment

The ultrasonic vocalizations of the rats were recorded during the manual therapy treatment of all rats, at least once per week using a recording system with ultralow-noise amplifiers designed for this purpose (Sonotrack, Metris, Hoofddorp, The Netherlands). Sonotrack software was used to analyze the number of calls, length of calls, and frequency of calls (in Hz) during the treatments. Mean results across weeks are reported. The individual performing the data analysis was naïve to group assignment.

### Examination of indices of potential discomfort during task performance

Indices of potential discomfort during task sessions were scored by at least two trained observers once per week (Fridays) using a check off observation sheet. This observation sheet included: sitting in the corner (during the entire session or for over 3 min during the active pulling time periods), fumbling, guarding, limb used to reach, and switching of the limb used to reach. The mean number of sessions in which a particular behavior was observed, per task day and per rat, is reported. The individual performing the data analysis was naïve to group assignment.

The limb(s) used to reach was recorded each session. TASK-Ac and TASK-Tx animals used in this study were ambidextrous beginning in week 2 and utilized either forearm to pull the lever bar interchangeably across each session, day, and week.

### Place preference test of cold temperature sensitivity

Cold temperature sensitivity was assessed using described methods [10, 34], once, a few days before euthanasia (to avoid the confounds of learning the test) using a two-temperature choice apparatus (T2T system, Bioseb, Marseille, France). On this instrument, rats were timed for how long they preferred to stand on a thermal plate set at room temp (22 °C), as opposed to a second plate of decreasing temperature (22 - 14 °C), in 2 °C steps (3 min per temperature step). A motion sensitive camera tracked the number of crossings between the two plates. The percent time spent on the variable plate at each temperature step, versus total time, was calculated from the system’s software.

### Reflexive maximum grip strength assay

All rats in the study underwent behavioral assays for reflexive grip strength, bilaterally, by a tester naïve to group assignment. This was tested using a rat grip strength meter (Columbus Instruments, Columbus, Ohio, United States), using described methods [7, 10]. The maximum reflexive grip strength achieved in 5 trials/limb is reported. Results are presented in centiNewtons (cN).

### Collection of serum and forearm flexor digitorum muscles for biochemical assay

Animals were deeply anesthetized with 5% isoflurane in oxygen, which is in accordance with *AVMA Guidelines for the Euthanasia of Animals*. Depth of anesthesia was assessed and monitored by the pattern and rate of respiration; the absence of muscle tone; and the absence of toe pinch reflex, tail pinch reflex, and eye blink reflex. When the animals no longer showed any reflexive responses, an absence of muscle tone, and breathing had halted, the animals underwent a thoracotomy and blood was then collected from the heart using cardiac puncture with a 23-gauge needle. This took place at 36 h after the final task session was completed in task week 10 in order to avoid possible serum cytokine fluctuations induced by exercise [36–39]. The blood was stored on ice for ~ 1 h until it clotted before being centrifuged for 20 min at 1000 g at 4^o^ C. Serum (the supernatant) was then collected and stored at -80^o^ C until assayed. From one limb per rat of the TASK groups (*n* = 10 per group) and C groups (*n* = 5 per group), mid-to proximal regions of the flexor digitorum muscles were separated from bones and then tendons with a scalpel, rinsed in sterile saline, dissected into smaller samples of about 50–100 mg each, and flash frozen in liquid nitrogen before storage at -80 °C, as previously described [7, 40]. A randomization scheme was used so that a mix of right and left limbs was collected for each assay choice (biochemical versus histology, a strategy used previously [11, 35], since TASK rats use both limbs for reaching).

### ELISA and Western blot assays

Unfixed and frozen muscle samples were collected and homogenized for protein assays, using described methods [40, 41]. Briefly, muscle samples were homogenized in sterile, ice-cold, phosphate-buffered saline (PBS) containing fresh proteinase inhibitors (cOmplete EDTA free Protease Inhibitor tablets, 5,056,489,001, Sigma-Aldrich, Inc., St. Louis, MO, United States). Homogenates were centrifuged at 12000 rpm for 15 min at 4 °C. Supernatants were aliquoted and stored at -80 °C until assayed via ELISA, in duplicate, for collagen type 1 (LS-F5638, LifeSpan BioSciences, Inc., Seattle, WA, United States) and transforming growth factor beta 1 (TGFβ-1; ADI-900-155, Enzo Life Sciences, Inc., Farmingdale, NY, United States), using manufacturers’ protocols. Muscle lysates were also assayed using a customized multiplex bead-based ELISA kit (LXSARM, R&D Systems) for IL-1β, IL-10, CCL2/MCP-1 and IL-18. Data (pg of protein) were normalized to μg total protein, determined using a bicinchoninic acid protein assay kit (23,227, BCA Protein assay, Pierce, ThermoFisher Scientific, Rockford, IL, United States). Serum was assayed for TNF-α levels (EA100366, Origene Technologies, Inc., Rockville, MD, United States), and is presented as pg protein per ml serum.

Aliquots of muscles lysates (equal amounts of protein) were assayed using Western blot methods for pro-collagen type I, using described methods [41]. Briefly, protein content was measured as above and equal amounts of protein per well were separated on 4–12% Tris-glycine gels (Wedgewell, XP04120BOX, Invitrogen, Carlsbad, CA, United States) without the presence of sodium dodecyl sulfate (SDS) in the gel itself, yet with 10% SDS in the running and sample buffers. Samples were boiled with BME in the sample buffer. The gels were run at 225 V for ~ 35 min. Purified rat tail collagen was used a molecular weight marker for collagen type I (A10493–01, Gibco, FisherScientific, United States), as was an iBright prestained protein ladder (LC5615, Invitrogen, Waltham, MA, United States). Gels were blotted onto nitrocellulose membranes (2 h at 20 V). Membranes were stained with Ponceau red to assess the consistency of loading, imaged, and then washed. Membranes were then blocked for 1 h with Odyssey Intercept buffer (927–70,001, LI-COR, Lincoln, NE, United States) with 1% donkey serum, at room temperature, and then incubated anti-collagen type I (ab6306, Abcam, Cambridge, MA, United States), diluted 1:1000, overnight at 4 °C with rocking. Membranes were washed in Tris buffered saline (TBS) with 0.05% Tween, and then incubated for 1 h with a donkey anti-mouse antibody conjugated to an 800 MW tag (IR-Dye 800, LI-COR) diluted 1:40,000. Membranes were imaged on an Odyssey Classic Infrared Imaging System (980–11,174, LI-COR). Gels and blots were repeated 3 times using different samples.

### Collection of forearm tissues for immunohistochemical and histological assays

Histological data were generated from the other limb from each animal in the TASK groups (*n* = 10 per group) and C groups (*n* = 5 per group). For this, after deep anesthetization and collection of muscles from one limb each rat for biochemical assays, as described above, the rats underwent intracardial perfusion with first saline and then buffered 4% paraformaldehyde. After fixation, the flexor digitorum muscle and tendon mass with the median nerve still intact through the forepaw, was separated from forelimb bones with a scalpel. A 2.5 mm thick cross-sectional piece of the flexor digitorum muscle was removed from the mid- to proximal elbow region for cross-sectioning, while the remaining mid-forarm to forepaw soft tissues were kept intact for longitudinal sectioning, as previously described [15, 35]. These pieces were immersion fixed for 48 h in buffered 4% paraformaldehyde before being cryopreseserved in 10% and then in 30% sucrose in phosphate buffer (48 h per sucrose solution). Tissues were embedded on dry ice in Optimum Cutting Temperature compound (23,730,571, FisherScientific, Houston, TX, United States) and cryosectioned at 14 μm (cross-sectionally for the proximal to mid- to proximal flexor digitorum muscle mass, and longitudinally for the remaining mid- to distal muscle-tendon-nerve mass). Sections were placed onto charged slides (22,037,200, FisherScientific, Pittsburgh, PA, United States), dried overnight at room temperature, before storage in foil-wrapped slide boxes at -80 °C until use.

Subsets of cryosections were stained in batched sets with specific antibodies against CD68 (a marker of primarily M1 type activated tissue macrophages in rats [42–44], ab31630, Abcam) at 1:300 dilution in PBS, CD206/mannose receptor (a marker of fibrogenic M2a type macrophages [31, 32]), ab64693, Abcam) at 1:500 dilution in PBS, and collagen type I (ab6308, Abcam, 1:300 dilution in PBS). The tissue sections, on slides, to be immunostained for CD68 or collagen type I first underwent 0.5% pepsin digestion in 0.01 N HCL for 15 min at room temperature, and then a 30 min incubation in 10% goat serum. The tissue sections, on slides, to be immunostained for CD206 first underwent 0.1% Tween20 incubation for 20 min at room temperature, and then a 30 min incubation in 10% goat serum. Sections were then incubated with one of the primary antibodies for overnight at room temperature. The sections were then washed in PBS (3 times, 5 min each), followed by incubation with the appropriate secondary antibody conjugated to green or red fluorescent tags (AF488 or Cy3; Jackson ImmunoResearch, West Grove, PA, United States) at a dilution of 1:100 each for 2 h at room temperature. DAPI was used as a nuclear counterstain following the immunostaining. The CD68 antibody used was previously validated using immunohistochemical methods [10]. The collagen type I antibody used was validated by Western blot (see Results section). The CD206/mannose receptor antibody was validated by leaving out the primary antibody (no nonspecific staining by the secondary was observed) and by comparing TASK rat results to C rat results.

Subsets of adjacent sections also underwent hematoxylin (Harris Modified, HHS, Sigma-Aldrich, St Louis, MO, United States) and light staining with aqueous eosin Y solution (HT1102, Sigma-Aldrich) (H&E staining) followed by coverslipping with 80% glycerol in phosphate buffer (to prevent tissue shrinkage), or Masson’s Trichrome staining followed by dehydration through increasing concentration of ethanol, cleared with xylene, and then coverslipped with DPX mountant (06522, Sigma-Aldrich).

### Image analysis

The individual performing the image analysis were naïve to group assignment. Numbers of CD68+ and CD206+ cells were counted in the median nerve at the level of the wrist, mid-muscle cross-sections of the flexor digitorum muscle, and in the flexor digitorum tendon, in at least 3 randomly chosen non-adjacent fields per tissue and per rat. This was performed using a motorized stage and a computerized image analysis system (Bioquant Life Science, Nashville, TN, United States) interfaced with a Nikon E800 epifluorescent microscope (Nikon, Melville, NY, United States) and a digital camera (R*etiga 4000R QImaging Firewire Cameras, Surry, BC Canada).* Numbers of immunostained cells were normalized to mm^2^ of the region of interest chosen for quantification. Extraneural collagen deposition around nerves was quantified in Masson’s Trichrome stained slides, while collagen type I immunofluorescence staining in muscles was quantified using previously described methods [13, 40].

Hematoxylin stained (with light eosin co-staining) flexor digitorum tendon sections were examined by two naïve examiners for histopathological changes (with results averaged). Proximal and mid/distal tendon regions were scored separately in one forelimb per rat using a previously described semi-quantitative scoring method (Bonar scale) [15, 45]. Tendon proper (interior) regions were scored for cell shape, cellularity, collagen organization, and presence of immune cells. Epitendon regions were scored for thickness, cellularity, and presence of immune cells. Epitendon and tendon vascularization was also scored for use in another study focused on angiogenic findings. Hematoxylin stained (with light eosin co-staining) stained sections containing tendons, muscles and nerves were also examined presence of neutrophils.

### Statistical analyses

GraphPad Prism version 8.2 (GraphPad, San Diego, CA, United States) was used for the statistical analyses. Significance was set at *p* = 0.05 and adjusted *p* values are reported. Results are reported as mean ± 95% confidence intervals (CI).

An a priori power analysis was performed using data from our prior studies on grip strength, CD68+ cells in muscles, and collagen type I protein levels [13, 34, 40]. We chose the most conservative sample size needed to detect differences at an alpha level of 0.05 and 80% power. Specifically, we found in those prior studies that for reflexive grip strength, sample sizes of 5 C and 9 TASK rats (each untreated) gave an effect size of 2.32, and that sample sizes of 9 untreated TASK and 6 TASK-Tx rats gave an effect size of 1.84. For numbers of CD68 immunopositive cells in tissues, sample sizes of 5 C and 5 TASK rats (each untreated) gave an effect size of 7.29, and sample sizes of 5 untreated TASK and 5 TASK-Tx rats gave an effect size of 5.93. For collagen type I protein levels, sample sizes of 5 C and 5 TASK rats (each untreated) gave an effect size of 2.13, and sample sizes of 5 TASK (untreated) and 5 TASK-Tx rats gave an effect size of 1.76. The power reached for each analysis was over 80% using these sample sizes. Therefore, in this study, C limbs were collected bilaterally, with one limb used for the ELISAs and the other for histological/immunohistological assays, providing *n* = 5 for both C groups for these assays. Since the TASK (TASK-Tx and TASK-Ac) animals show variability in learning, discomfort or tissue responses, we increased their number to *n* = 10/group/assay.

Next, data was tested for Normality using Shapiro-Wilk and Kolmogorov-Smirnov tests of normality, and residuals were inspected. The data was normally distributed for each assay. Thereafter, two-way ANOVAs were used to analyze the ultrasound data and number of cells per mm^2^ of tissue using the factors: treatment region (manual therapy treatment of upper extremities (Tx) versus lower extremities (active control, Ac), and group (TASK versus C). These were followed by Tukey’s post hoc multiple comparisons tests to detect between group differences. Linear mixed-effects models with repeated measures (REML method, Restricted Maximum Likelihood) with Geisser-Greenhouse corrections were used to assay reflexive grip strength and spontaneous behaviors indicative of discomfort results using the factors: treatment region, group, and week. A linear mixed-effects model with repeated measures was also used to assay cold temperature sensitivity using the factors: treatment region, task group, and temperature. These were followed by Dunnett’s post hoc multiple comparisons tests to determine between group differences. F ratios as an indication of the robustness of the results, main and interaction effects, and their *p* values are reported in Tables 1 and 2. Significant post hoc results are indicated in the figure panels.

**Table 1:** Two-way ANOVA and mixed-effects model results for behavioral changes

**Sonotrack Vocalization,** means across weeks assayed using 2-way ANOVAs			
	*Treatment*	*Group (TASK* vs *C)*	*Significant Interactions*
Mean # calls	*F*_(1,25)_ = 0.21, *p* = 0.65	*F*_(1,25)_ = 0.15, *p* = 0.70	None
Mean length of calls	*F*_(1,24)_ = 0.21, *p* = 0.54	*F*_(1,24)_ = 0.008, *p* = 0.87	None
Mean frequency of calls	*F*_(1,24)_ = 0.42, *p* = 0.54	*F*_(1,24)_ = 1.20, *p* = 1.23	None
**Cold Temperature Sensitivity,** assessed in Week 12, assayed using a linear mixed-effects model			
*Treatment*	*Group*	*Temperature*	*Significant Interactions*
*F*_(1,72)_ = 7.91, *p* = 0.006	*F*_(1,80)_ = 10.50, *p* = 0.002	*F*_(4,80)_ = 8.84, *p* < 0.0001	Treatment x Group: *F*_(1,72)_ = 6.75, *p* = 0.01
**Reflexive Grip Strength,** assessed every 3 weeks, assayed using a mix-effects model with repeated measures			
*Treatment*	*Group*	*Week*	*Significant Interactions*
*F*_(1,49)_ = 10.93; *p* = 0.002	*F*_(1,49)_ = 6.83; *p* = 0.01	*F*_(4,190)_ = 0.93, *p* = 0.45	Treatment x Group: *F*_(1,49)_ = 4.98, *p* = 0.03
**Sits in Corner/ Task Avoidance,** assessed every 3 weeks, assayed using a mix-effects model with repeated measures			
*Treatment*	*Group*	*Week*	*Significant Interactions*
*F*_(1,36)_ = 5.02, *p* = 0.03	N/A	*F* (5, 48) = 1.27, *p* = 0.29	None

**Table 2:** Two-way ANOVA results for protein and immuno/histochemical assays

Outcome	Tissue	Treatment Region (UE vs LE)	Group (Task vs C)	Treatment Region x Group Interaction
**CD68+ cells/mm**^**2**^	Median Nerve	*F*_(1,26)_ = 5.78, *p* = 0.02	*F*_(1,26)_ = 5.74; *p* = 0.02	*F*_(1,26)_ = 5.62; *p* = 0.03
**“**	Flexor digitorum muscle	*F*_(1, 26)_ = 17.87, *p* = 0.0003	*F*_(1, 26)_ = 35.85; *p* < 0.0001	*F*_(1, 26)_ = 15.04; *p* = 0.0006
**“**	Flexor digitorum tendon	*F*_(1, 26)_ = 17.36, *p* = 0.0003	*F*_(1, 26)_ = 17.36; *p* = 0.0003	*F*_(1, 26)_ = 15.39; *p* = 0.0006
**CD206+ (Mannose Receptor+) cells/mm**^**2**^	Median Nerve	*F*_(1,26)_ = 3.96, *p* = 0.057	*F*_(1,26)_ = 9.16, *p* = 0.005	*F*_(1,26)_ = 2.87, *p* = 0.10
**“**	Flexor digitorum muscle	*F*_(1, 26)_ = 6.50; *p* = 0.01	*F*_(1, 26)_ = 36.14; *p* < 0.0001	*F*_(1, 26)_ = 4.75; *p* = 0.04
**“**	Flexor digitorum tendon	*F*_(1, 26)_ = 12.91; *p* = 0.001	*F*_(1, 26)_ = 19.55; *p* = 0.0002	*F*_(1, 26)_ = 14.66; *p* = 0.0007
**Inflammatory Cytokines (pg/μg total protein)**				
IL-1β	Flexor digitorum muscle	*F*_(1, 22)_ = 3.82, *p* = 0.06	*F*_(1, 22)_ = 7.49, *p* = 0.01	*F*_(1, 22)_ = 3.39, *p* = 0.08
IL-10	**“**	*F*_(1, 21)_ = 8.07, *p* = 0.009	*F*_(1, 21)_ = 11.48, *p* = 0.003	*F*_(1, 21)_ = 8.32 *p* = 0.009
IL-18	**“**	*F*_(1, 22)_ = 0.35, *p* = 0.56	*F*_(1, 22)_ = 0.38, *p* = 0.54	*F*_(1, 22)_ = 0.02, *p* = 0.89
CCL2/MCP-1	**“**	*F*_(1, 22)_ = 0.006, *p* = 0.94	*F*_(1, 22)_ = 7.19, *p* = 0.94	*F*_(1, 22)_ = 0.66, *p* = 0.43
TNF-α	Serum	*F*_(1, 26)_ = 0.30, *p* = 0.59	*F*_(1, 26)_ = 0.73, *p* = 0.40	*F*_(1, 26)_ = 01.22, *p* = 0.28
**Extraneural Collagen** % collagen staining	Median Nerve	*F*_(1, 26)_ = 13.97; *p* = 0.0009	*F*_(1, 26)_ = 37.16; *p* < 0.0001	*F*_(1, 26)_ = 12.47; *p* = 0.002
**Collagen Type I**				
% immunostaining	Flexor digitorum muscle	*F*_(1, 26)_ = 17.02, *p* = 0.0003	*F*_(1, 26)_ = 45.53, *p* < 0.0001	*F*_(1, 26)_ = 18.05, *p* = 0.0002
pg/μg total protein	**“**	*F*_(1, 26)_ = 5.93, *p* = 0.024	*F*_(1, 26)_ = 4.34, *p* = 0.047	*F*_(1, 26)_ = 4.96; *p* = 0.04
**Transforming Growth Factor beta 1 (TGF-β1)** pg/μg total protein	Flexor digitorum muscle	*F*_(1,26)_ = 0.10, *p* = 0.75	*F*_(1,26)_ = 0.22.74, *p* < 0.0001	*F*_(1,26)_ = 0.15, *p* = 0.70
**Mid/Distal Flexor Digitorum Tendon Pathology Scores**				
Endotendon cell shape	Mid/Distal flexor digitorum tendon	*F*_(1,26)_ = 3.94, *p* = 0.06	*F*_(1,26)_ = 47.21, *p* < 0.0001	*F*_(1,26)_ = 7.18, *p* = 0.01
Endotendon cellularity	**“**	*F*_(1,26)_ = 9.90, *p* = 0.004	*F*_(1,26)_ = 22.10, *p* < 0.0001	*F*_(1,26)_ = 4.18, *p* = 0.05
Endotendon collagen organization	**“**	*F*_(1,26)_ = 3.93, *p* = 0.049	*F*_(1,26)_ = 3.50, *p* = 0.07	*F*_(1,26)_ = 0.52, *p* = 0.48
Endotendon immune cells	**“**	*F*_(1,26)_ = 0.84, *p* = 0.37	*F*_(1,26)_ = 2.80, *p* = 0.11	*F*_(1,26)_ = 0.84, *p* = 0.37
Epitendon thickness	**“**	*F*_(1,26)_ = 4.11, *p* < 0.0001	*F*_(1,26)_ = 43.22, *p* = 0.05	*F*_(1,26)_ = 1.16, *p* = 0.29
Epitendon cellularity	**“**	*F*_(1,26)_ = 16.54, *p* = 0.09	*F*_(1,26)_ = 3.08, *p* = 0.0004	*F*_(1,26)_ = 2.15, *p* = 0.15
Epitendon immune cells	**“**	*F*_(1,26)_ = 4.61, *p* = 0.04	*F*_(1,26)_ = 18.14, *p* = 0.0002	*F*_(1,26)_ = 3.14, *p* = 0.09

## Results

### Comparison of forces delivered to forelimb

The forces delivered to the forelimb during the three modalities provided (Fig. 1b-d) showed similar peak forces to the added two modalities previously used (Fig. 1e and f). When applied directly to the forelimb, imaging showed that forces of 100 cN and more almost fully compressed the forelimb flexor structures (Fig. 1g and h).

### The manual therapy treatment used was well tolerated by the animals

An ultrasonic recording system was used to assess whether the manual therapy treatment was tolerated. No significant differences between the four groups were observed in the mean number of calls (Table 1 and Supplemental Fig. 2). Thus, all TASK and C groups similarly tolerated treatment of either their forelimbs or hindlimbs.

### Upper extremity manual therapy reduced task-induced increases of CD68+ macrophages in forelimb nerves, muscles and tendons

**Fig. 2:**
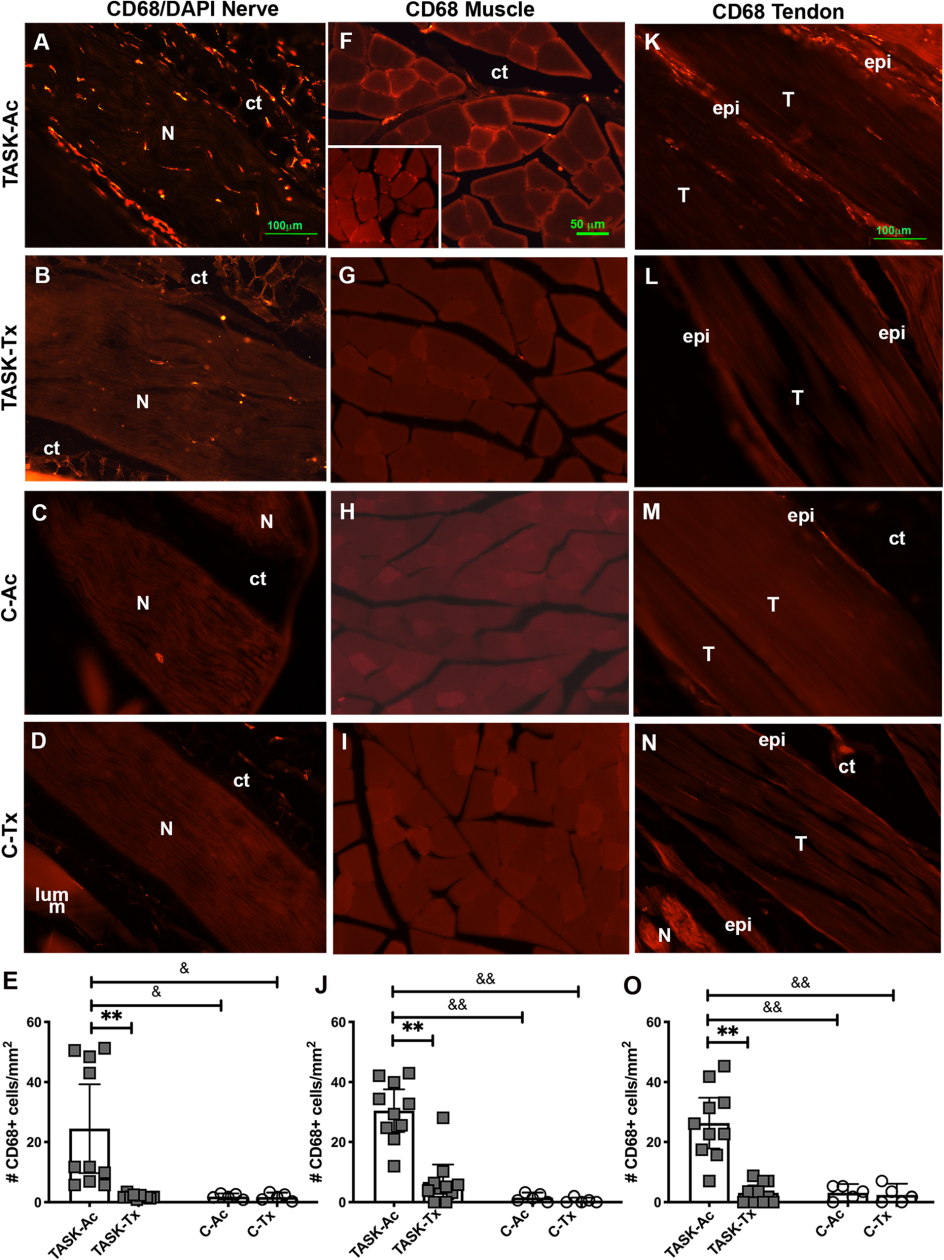
Images and quantification of CD68-immunopositive macrophages (stained red) in forearm tissues. Treated (TASK-Tx and C-Tx) animals received manual therapy to their upper extremities, bilaterally, while active control (TASK-Ac and C-Ac) animals received manual therapy to their lower extremities, bilaterally. **a**-**d** CD68+ cells in longitudinal sections of median nerves at the level of the wrist. **e** Numbers of CD68+ cells in median nerves at the level of the wrist. **f**-**i** CD68+ cells in flexor digitorum muscles, in cross-sections from mid-forearm regions. **j** Numbers of CD68+ cells per mm^2^ in flexor digitorum muscles, in cross-sections from mid-forearm regions. **k**-**n** CD68+ cells in longitudinal sections of flexor digitorum tendons, in mid- to distal regions. **o** Numbers of CD68+ cells per mm^2^ in flexor digitorum tendons, in mid- to distal regions. Abbreviations: ct = connective tissue, epi = epineurium in nerves and epitendon in tendons; N = median nerve; T-tendon. Scale bar in panel **a** = 100 μm and applies to panels **b**-**d**, **k**-**n**. Scale bar in panel **e** = 50 μm and applies to panels **f**-**i**. Symbols in **e**, **j** and **o** **: *p* < 0.01, compared to TASK-Tx group; ^&^: *p* < 0.05 and ^&&^: *p* < 0.01, compared to a control group as shown. Mean ± 95% CI shown

We and others have shown that enhanced sensitivity and discomfort is related to nerve inflammation [12, 46, 47], and that reflexive grip strength declines can be related to flexor digitorum muscle, tendon sheath inflammation, and median neuropathies [10, 19, 46, 48–50]. Since CD68 is a marker of inflammatory M1 type macrophages in rats [42, 51], we examined CD68 immunopositive (+) inflammatory cells in several forearm tissues used to reach. Microscopic examination showed more CD68+ cells within and around median nerves of TASK-Ac rats, relative to TASK-Tx, C-Ac and C-Tx rats (Fig. 2a-d). Similar elevation of CD68+ cells was observed in flexor digitorum muscles (in endomyseal connective tissues surrounding myofibers) of TASK-Ac rats, relative to the other groups (Fig. 2f-i). More CD68+ cells were observed in flexor digitorum tendons (primarily in epitendons) of TASK-Ac rats, relative to relative to the other groups (Fig. 2k-n).

Numbers of CD68+ cells per mm^2^ were quantified in each tissue. In median nerves at the level of the wrist, a two-way ANOVA showed significant effects for treatment region, group, and their interaction (*p* = 0.02, *p* = 0.02, and *p* = 0.03, respectively, Table 2). Post hoc analysis showed more CD68+ cells in the median nerves of TASK-Ac rats, compared to the other groups (Fig. 2e). In mid- to proximal flexor digitorum muscles, a two-way ANOVA showed significant effects for treatment region, group, and their interaction (*p* = 0.0003, *p* < 0.0001, and *p* = 0.0006, respectively, Table 2). Post hoc analysis showed higher numbers of CD68+ cells in muscles of TASK-Ac rats, compared to the other groups (Fig. 2j). In flexor digitorum tendons, a two-way ANOVA showed significant effects for treatment region, group, and their interaction (*p* = 0.0003, *p* = 0.0003, and *p* = 0.0006, respectively, Table 2). Post hoc analysis showed higher numbers of CD68+ cells in the tendons of TASK-Ac rats, compared to the other groups (Fig. 2o). In each tissue, results in TASK-Tx rats did not differ from the results of either C group (Fig. 2e, j and o).

### Upper extremity manual therapy reduced task-induced increases of CD206+ macrophages in forelimb nerves, muscles, and tendons

**Fig. 3:**
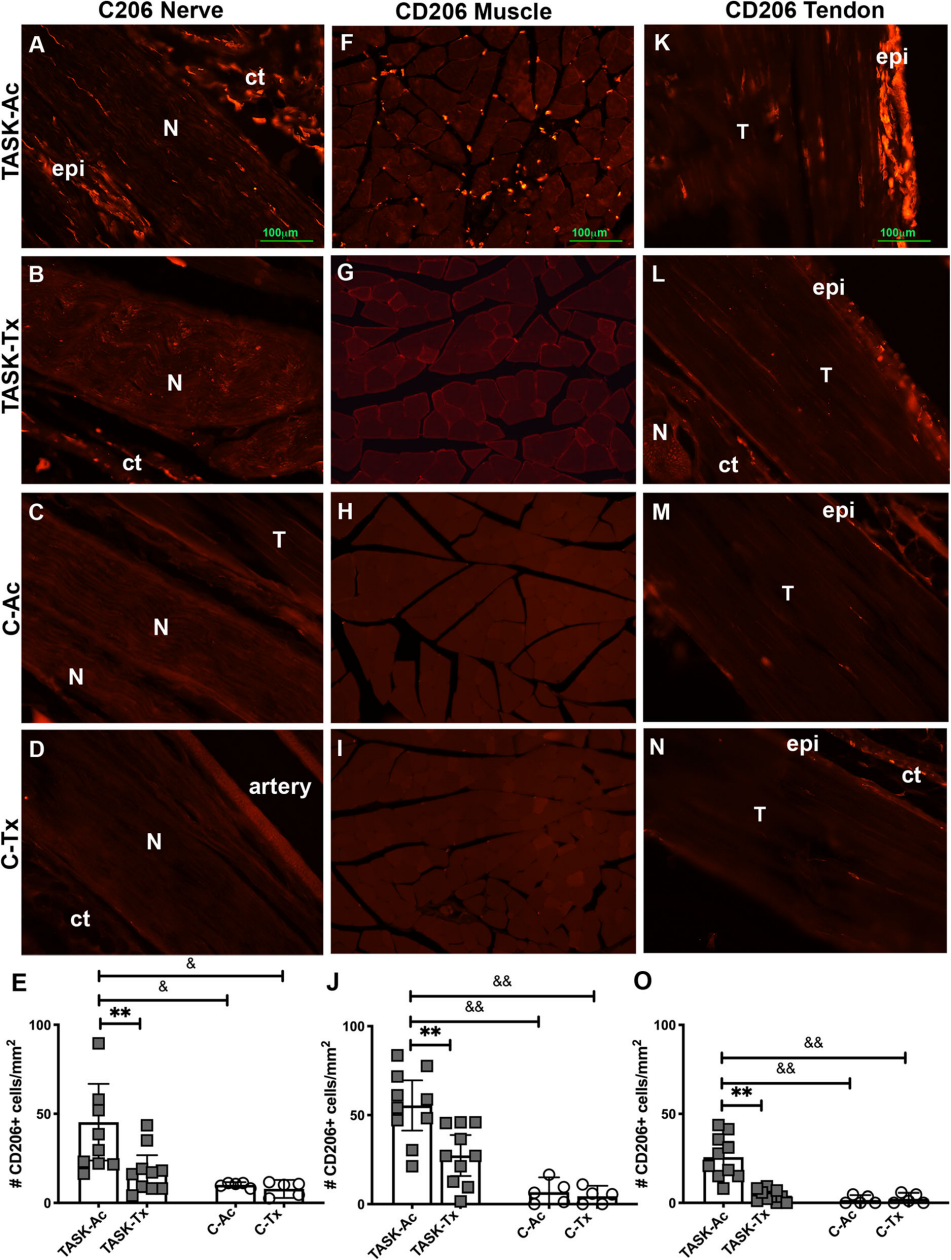
Images and quantification of CD206 (mannose receptor) immunopositive (+) macrophages (stained red) in forearm tissues. **a**-**d** CD206+ cells in longitudinal sections of median nerves at the level of the wrist. **e** The number of CD206+ cells per mm^2^ in the median nerve at the level of the wrist. **f**-**i** CD206+ cells in cross-sections of mid-forearm regions of flexor digitorum muscles. **j** The number of CD206+ cells per mm^2^ in flexor digitorum muscles, in cross-sectional mid-forearm regions. **k**-**n** CD206+ in longitudinal sections of flexor digitorum tendons. **o** The number of CD206+ cells per mm^2^ in flexor digitorum tendon, in mid- to distal regions. Abbreviations: ct = connective tissue, epi = epineurium in nerves and epitendon in tendons; N = median nerve, T = tendon. Scale bar in panels **a**, **f** and **k** = 100 μm and applies to all other panels. Symbols in **e**, **j** and **o** **: *p* < 0.01, compared to TASK-Tx group; ^&^: *p* < 0.05 and ^&&^: *p* < 0.01, compared to a control group as shown. Mean ± 95% CI shown

Since CD206, a mannose receptor, is a marker of fibrogenic M2a type macrophages in rats [31, 32], we examined for CD206+ macrophages in several forearm tissues used to reach. Microscopic examination showed more CD206+ cells within and surrounding median nerves in TASK-Ac rats (mainly in the epineurium), relative to TASK-Tx, C-Ac and C-Tx rats (Fig. 3a-d). Similar higher numbers of CD206+ cells were observed in flexor digitorum muscles (in endomyseal connective tissues surrounding the myofibers) of TASK-Ac rats, relative to the other groups (Fig. 3f-i). More CD206+ cells were also observed in flexor digitorum tendons (primarily in the epitendons) of TASK-Ac rats, relative to the other groups (Fig. 3k-n).

Numbers of CD206+ cells per mm^2^ were quantified in each tissue. In the median nerve at the level of the wrist, a two-way ANOVA showed a significant effect for group (*p* = 0.005, Table 2) and a strong trend towards a treatment region effect (*p* = 0.057, Table 2). Post hoc analysis showed more CD206+ cells in the median nerve of TASK-Tx rats, compared to the other groups (Fig. 3e). In the mid- to proximal flexor digitorum muscle, a two-way ANOVA showed significant effects for treatment region, group, and their interaction (*p* = 0.01, *p* < 0.0001, and *p* = 0.04, respectively, Table 2). Post hoc analysis showed more CD206+ cells in muscles of TASK-Ac rats, compared to the other groups (Fig. 3j). In the flexor digitorum tendons, a two-way ANOVA showed significant effects for treatment region, group, and their interaction (*p* = 0.001, *p* = 0.0002, and *p* = 0.0007, respectively, Table 2). Post hoc analysis showed more CD206+ cells in these tendons of TASK-Ac rats, compared to the other groups (Fig. 3o). In each tissue, results in TASK-Tx rats did not differ from the results of either C group (Fig. 3e, j, and o).

### In muscles, upper extremity manual therapy decreased IL-1β and increased IL-10

**Fig. 4:**
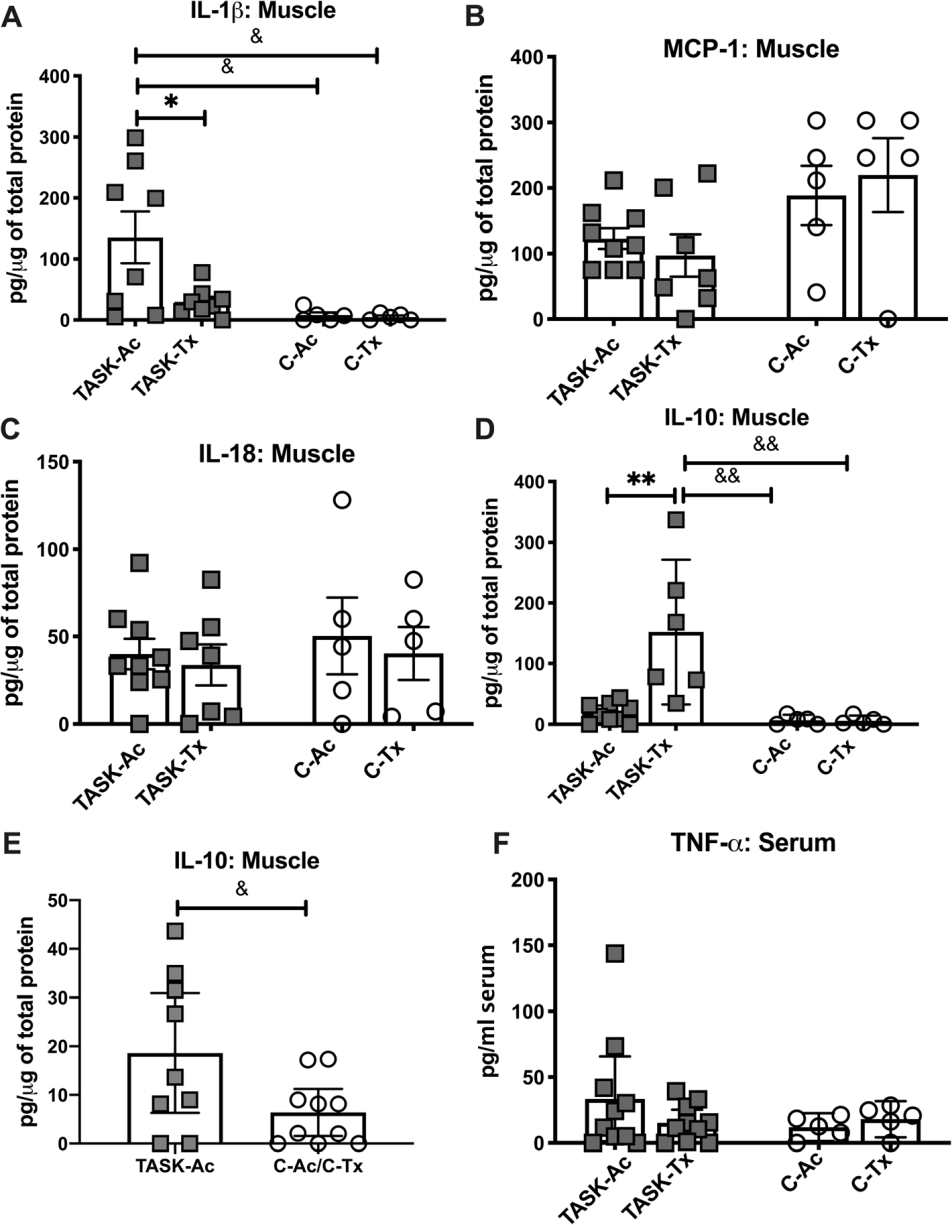
Pro-and anti-inflammatory cytokines assayed in flexor digitorum muscle lysates and serum using ELISA. **a** IL-1β in muscles. **b** CCL2/MCP-1 in muscles. **c** IL-18 in muscles. **d** IL-10 in muscles. **e** IL-10 in TASK-Ac muscles versus in C-Ac/C-Tx muscles (the latter data was combined for this analysis). **f** TNFα in serum. Symbols: *: *p* < 0.05 and **: *p* < 0.01, compared to TASK-Tx group; ^&^: *p* < 0.05 and ^&&^: *p* < 0.01, compared to a control group as shown. Mean ± 95% CI shown

Multiplex ELISAs showed group differences in muscle levels of IL-1β and IL-10, although not IL-18 and CCL2/MCP-1 levels (Fig. 4a-e; Table 2). Muscle levels of IL-1β, a pro-inflammatory cytokine, showed a significant effect for group (*p* = 0.01, Table 2). Post hoc analysis showed increased levels of IL-1β in TASK-Ac muscles, compared to the other groups (Fig. 4a). Muscle levels of IL-18 and CCL2/MCP-1 did not differ across groups (Table 2, Fig. 4b and c). In contrast to IL-1β, muscle levels of IL-10, a potent anti-inflammatory cytokine also showed significant effects for treatment region, group, and their interaction (*p* = 0.009, *p* = 0.003, and *p* = 0.009, respectively, Table 2). Post hoc analysis showed increased IL-10 in Task-Tx muscles, compared to the other groups (Fig. 4d) [30, 32]. When muscle levels of IL-10 in TASK-Ac rats were compared to the combined C groups’ results, a small yet significant difference was observed (Fig. 4e). Serum levels of TNF-α did not differ across groups (Fig. 4f).

### In median nerves, upper extremity manual therapy reduced task-induced epineurial thickening

**Fig. 5:**
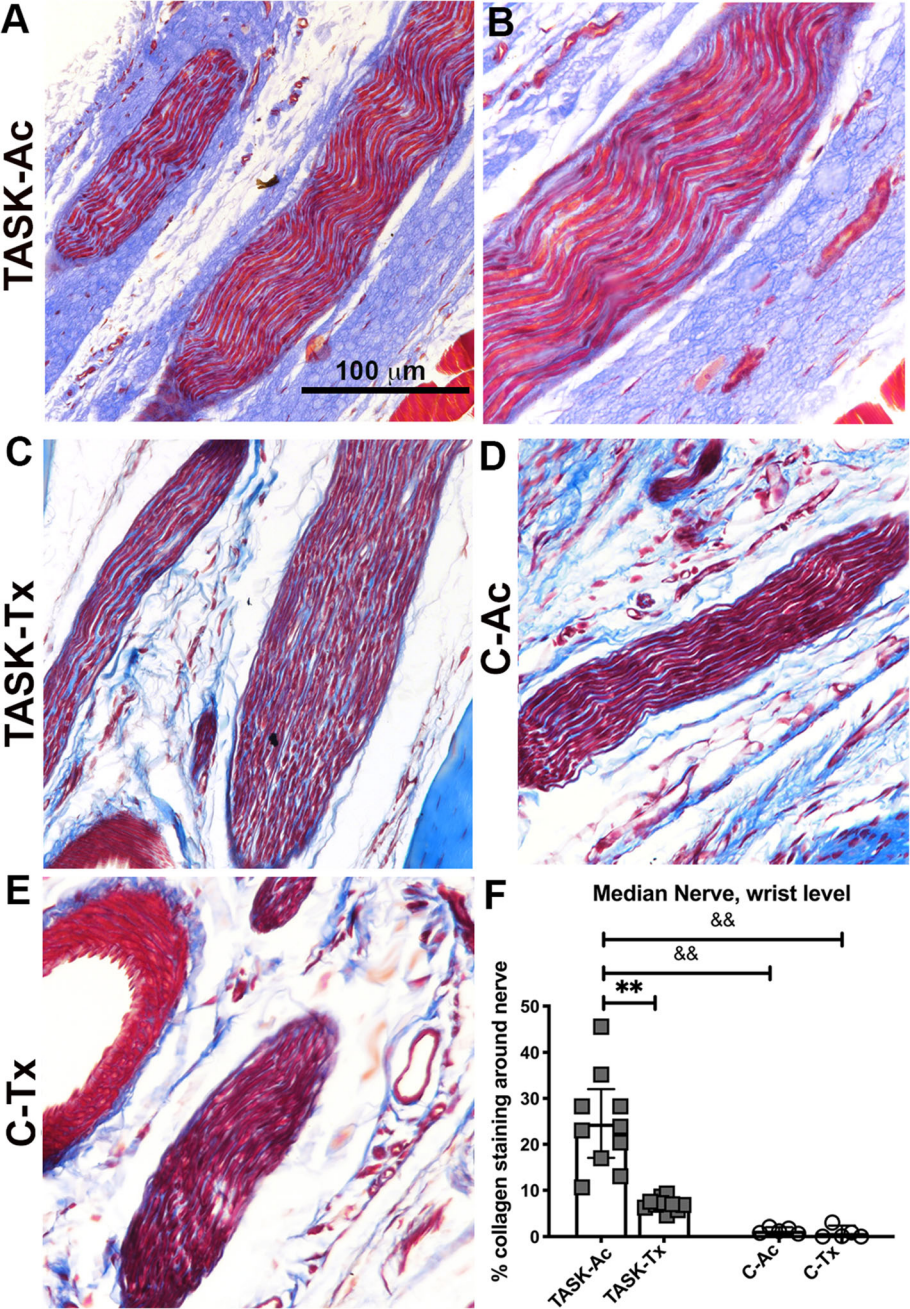
Masson’s Trichrome staining for collagen around median nerves at wrist level. **a**-**e** Representative images from each group. **f** Percent area with collagen staining around the median nerve. Scale bar in panel **a** = 100 μm and applies to the other panels. **: *p* < 0.01, compared to TASK-Tx group; ^&&^: *p* < 0.01, compared to a control group as shown. Mean ± 95% CI shown

Since we have previously shown that prolonged performance of this repetitive task can lead to epineurial thickening [12–14], and that our past more extensive manual therapy treatment combination prevented development of this fibrotic change [13], we next examined for such changes in this study. We observed significant thickening of the epineurium around median nerves at the level of the wrist in TASK-Ac rats, relative to TASK-Ac rats and each control rat group (Fig. 5a-e). A two-way ANOVA showed significant effects for treatment region, group and their interaction (*p* = 0.0009, *p* < 0.0001, and *p* = 0.002, respectively, Table 2). Post hoc analysis showed increased % collagen staining around the median nerve at the level of the wrist in TASK-Ac rats, compared to the TASK-Tx and C groups, and that the TASK-Tx group did not differ from the results of either C group (Fig. 5f).

### In flexor digitorum muscles, upper extremity manual therapy reduced task-induced fibrogenic changes

**Fig. 6:**
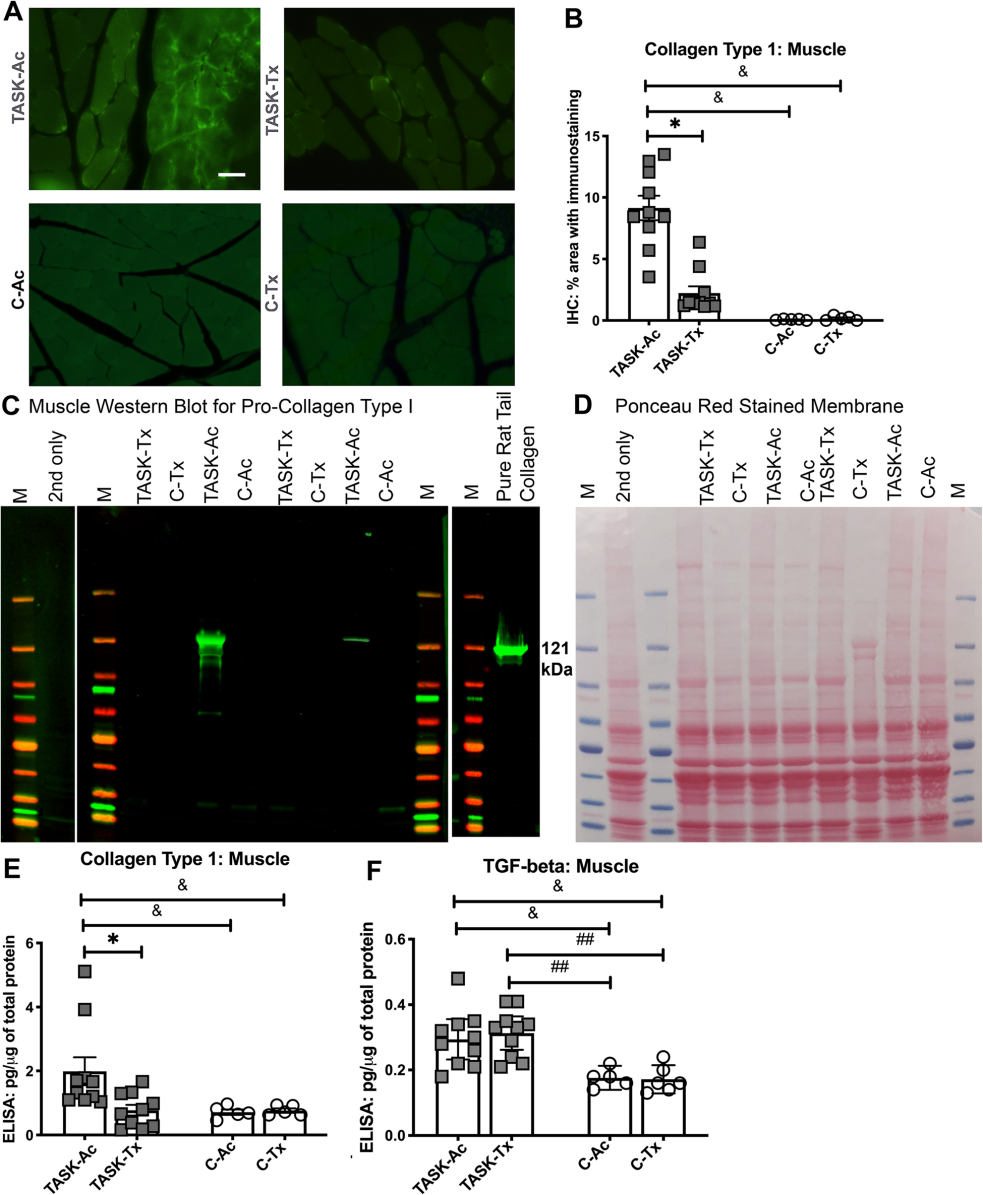
Collagen type I in forearm flexor digitorum muscles. **a** Representative images of muscle cross-sections immunostained for collagen type I. Scale bar in upper left panel = 50 μm and applies to each image. **b** Quantification of the collagen type I immunostaining, with percent area with collagen type I staining per mm^2^ shown. **c** Muscle lysates assayed using Western blot methods. M = denotes lanes loaded with a prestained protein ladder as a Marker. Left panel: Membrane strip with two lanes probed only with the 2nd antibody. Middle panel: Muscle lysate samples from two animals per group were loaded into lanes as shown. The membrane was probed with collagen type I antibody (using the same antibody as used for the immunohistochemistry in panel **a**). Right panel: Membrane strip with one lane loaded with purified rat tail collagen as a positive marker for collagen type I (~ 121 kDa). **d** The same membrane shown in Panel (**d**) stained with Ponceau red to show amount of protein loaded per lane. **e** Collagen type I levels in muscle lysates, quantified using ELISA. **f** Transforming growth factor β 1 (TGF-β1) levels in muscle lysates, quantified using ELISA. Symbols: *: *p* < 0.05, compared to TASK-Tx group; ^&^: *p* < 0.05 and ^&&^: *p* < 0.01, compared to a control group as shown. Mean ± 95% CI shown

Immunostaining of muscle cryosections for collagen type I from one limb per rat revealed more collagen deposition in endomyseal connective tissues around myofibers in TASK-Ac muscles, relative to TASK-Tx, C-Ac and C-Tx muscles (Fig. 6a). Quantification of that immunostaining and then a two-way ANOVA showed significant effects for treatment region, group and their interaction (*p* = 0.0003, *p* < 0.0001, and *p* = 0.0002, respectively, Table 2). Post hoc analysis showed that collagen type I immunostaining was higher in the TASK-Ac muscles, compared to the other three groups (Fig. 6b). Collagen type I changes in muscles of the contralateral limbs (also used to reach by TASK rats) were also observed using Western Blot analysis, using the same antibody as for the immunostaining (confirming the specificity of the antibody for pro-collagen type I in the muscles relative to the purified rat tail collagen results shown in the right panel of Fig. 6c). More procollagen type I was observed in TASK-Ac muscles (121 kDa), relative to the other groups (Fig. 6c), with Ponceau red staining of the membranes used to show similar protein loading in most lanes (Fig. 6d). Muscle levels of collagen type I protein were quantified using ELISA in other aliquots of the same muscle lysate samples (Fig. 6e). Two-way ANOVA showed significant effects for treatment region, group, and their interaction (*p* = 0.02, *p* = 0.047, and *p* = 0.04, respectively, Table 2). Post hoc analysis showed that collagen type I levels were higher in TASK-Ac muscles, compared to TASK-Tx and C-Ac muscles (Fig. 6e). Levels of TGFbeta 1 were also analyzed in muscle lysate samples using ELISA, and showed a significant group effect in the two-way ANOVA (p < 0.0001, Table 2). Post hoc analysis showed that TGF-β1 levels were higher in muscles of both TASK groups, compared to both C groups (Fig. 6f).

### In flexor digitorum tendons, upper extremity manual therapy reduced task-induced increases in tendinopathy

**Fig. 7:**
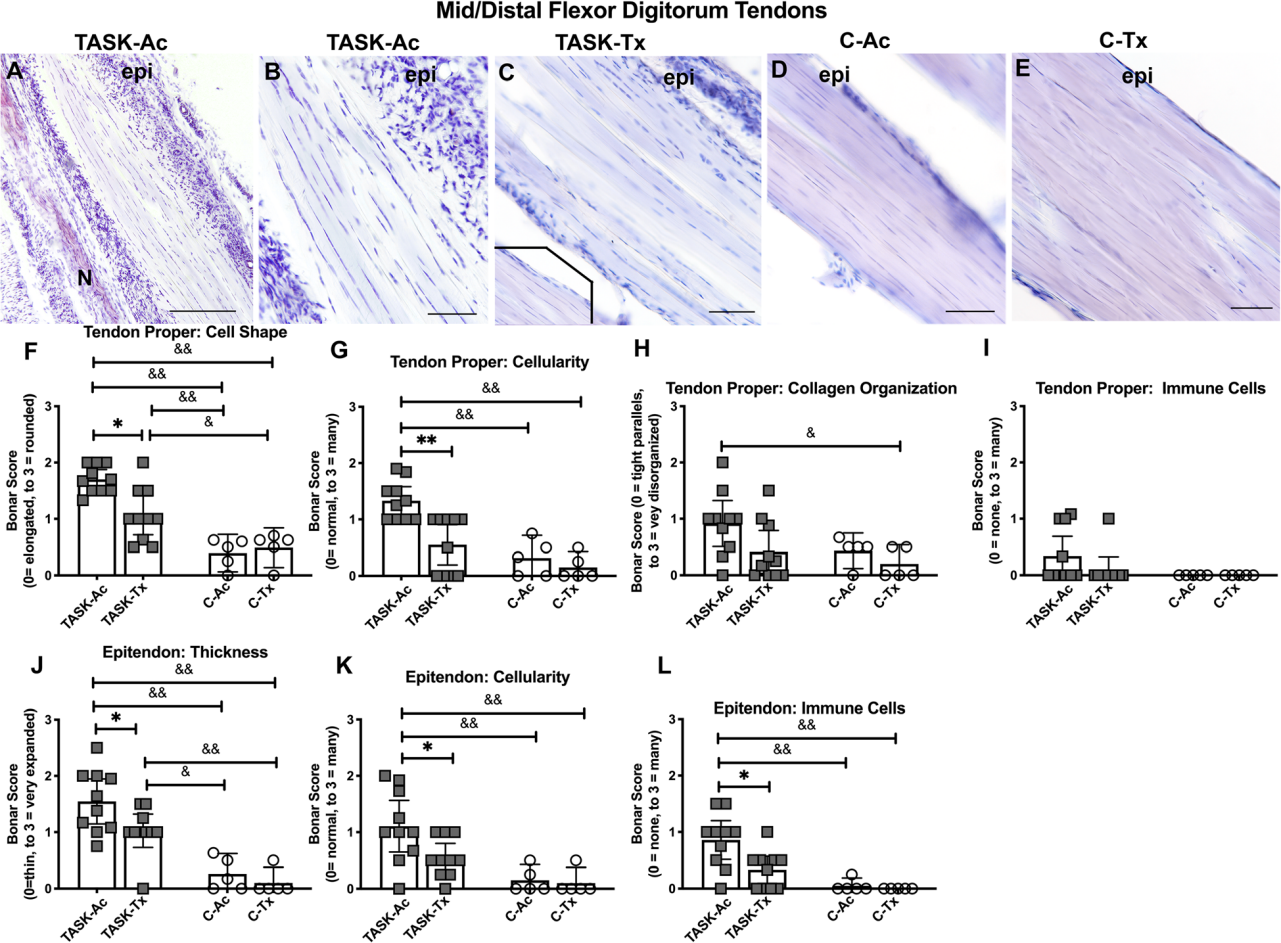
Images and histological changes in mid/distal regions of flexor digitorum tendons. A Bonar scoring system was used to rate the histological findings. **a**-**e** Representative images of forearm flexor digitorum tendons at the level of the wrist. Inset in Panel **c** shows a different TASK-Tx rat’s mid flexor digitorum tendon. **f**-**i** Tendon scores of cell shape, cellularity, collagen organization and presence of immune cells, respectively. **j**-**l** Epitendon of thickness, cellularity, and presence of immune cells. Scale bars in each image = 50 μm. Abbreviations: Epi = epitendon; N = a median nerve branch. Symbols: *: *p* < 0.05 and **: *p* < 0.01, compared to TASK-Tx group; ^&^: *p* < 0.05 and ^&&^: *p* < 0.01, compared to a control group as shown. Mean ± 95% CI shown

Since we have previously shown that prolonged performance of high intensity repetitive tasks induce pathological changes in flexor digitorum tendons [15, 52], we next examined for such changes in this study. We first examined for changes in mid- to distal flexor digitorum tendon proper (interior) regions and saw more rounded cells and moderately enhanced cellularity and collagen fibril disorganization in TASK-Ac tendons, relative to the other groups (Fig. 7a-e). Details of the two-way ANOVA findings are listed in Table 2. Post hoc analysis showed that tendon cell shape scores were higher in TASK-Ac mid/distal tendon regions, compared to the same region in TASK-Tx, C-Ac, and C-Tx groups, as well as in Task-Tx mid/distal tendon regions, compared to the same region in C-Ac and C-Tx groups (Fig. 7f). Scores for tendon cellularity were higher in TASK-Ac mid/distal tendons, compared to Task-Tx and both C groups (Fig. 7g). Scores for tendon collagen organization were higher in TASK-Ac mid/distal tendon regions, compared to C-Tx mid/distal tendon regions (Fig. 7h). There were no significant post hoc changes in immune-like cell scores across groups in mid/distal tendon regions (Fig. 7i).

We next examined for changes in epitendons of flexor digitorum mid/distal tendons and observed enhanced epitendon thickness, cellularity and immune-like cells in TASK-Ac tendons, relative to the other groups (Fig. 7a-e). Details of the two-way ANOVA findings are listed in Table 2. Post hoc analysis showed that epitendon thickness was higher in TASK-Ac mid/distal tendon regions, compared to the same regions in TASK-Tx, C-Ac, and C-Tx groups, as well as in Task-Tx mid/distal tendons, compared to C-Ac and C-Tx groups (Fig. 7j). Scores for epitendon cellularity as well as presence of immune-like cells in epitendon regions were higher in TASK-Ac mid/distal regions, compared to Task-Tx and C groups (Fig. 7k and l).

We also examined for changes in endo- and epitendons of proximal (intramuscular) regions of the flexor digitorum tendons and observed no significant changes (Supplemental Figure 3).

### No neutrophils were observed in any group’s tissues

Hematoxylin stained (with light eosin co-staining) stained sections containing median nerve branches, flexor digitorum muscles and tendons were also examined presence of neutrophils. None were noted in these tissues of any group, as shown in nerves in Supplemental Figure 4, in tendons in Fig. 7a-e, and as shown previously in muscles of 12 week TASK rats [15, 34].

### Upper extremity manual therapy prevented task-induced somatosensory and motor declines

**Fig. 8:**
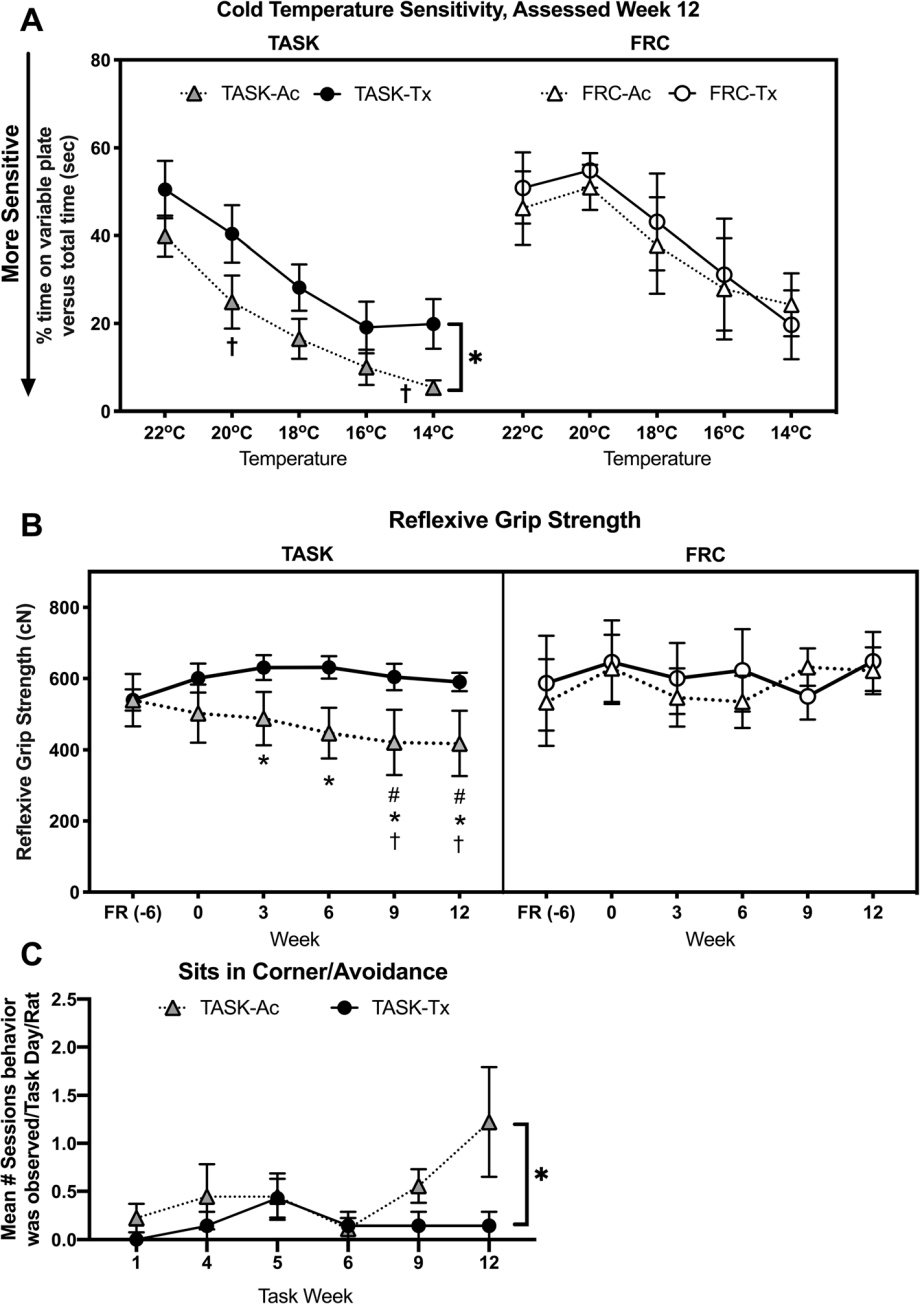
Sensorimotor changes across weeks. **a** Cold temperature sensitivity assayed using a temperature place preference instrument in the last week of task performed (week 12) in TASK rats, and at an age-matched time points in control rats. **b** Reflexive grip strength was assayed at baseline after onset of food restriction, after the 6-week shaping period (task week 0, and every 3 weeks through task week 12). Data are reported bilaterally for the TASK rats (TASK-Ac and TASK-Tx, *n* = 10/group) and control rats (C-Ac and C-Tx; *n* = 5 rats/group). **c** Observed incidence of sitting in the corner (suggestive of task avoidance) during an active task session rather than reaching and pulling for longer than 3 min. Symbols: *: *p* < 0.0, compared to TASK-Tx group; #: *p* < 0.05, compared to baseline; †: *p* < 0.05, compared to age- and treatment-matched control animals. Mean ± 95% CI shown

We have previously reported increased sensorimotor declines in long-term untreated TASK rats (such as, enhanced cold sensitivity, declines in reflexive grip strength, and spontaneous indices of discomfort during task performance) in parallel with both increased inflammatory and fibrogenic tissue changes [10, 11, 13]. Therefore, we next assessed these behaviors. Cold temperature sensitivity was assessed only in task week 12 to avoid the rats learning the outcomes of this test (Fig. 8a). A linear mixed-effects model with repeated measures for the multiple temperatures tested revealed significant effects for treatment region (*p* = 0.006), group (*p* = 0.002), temperature (*p* < 0.0001), and a treatment x group interaction (*p* = 0.01, Table 1). Post hoc comparisons showed that TASK-Ac rats were more sensitive to 14 °C, compared to the other three groups, and that TASK-Tx rats did not differ from either C group (Fig. 8a).

Regarding forearm reflexive grip strength, linear mixed-effects modeling with repeated measures for multiple testing across weeks, revealed significant effects for treatment region, group and their interaction (*p* = 0.002, *p* = 0.01, and *p* = 0.03, respectively, Table 1). Post hoc analysis showed that TASK-Ac rats had decreased reflexive grip strength in task weeks 3 through 12, compared to TASK-Tx rats, and in task weeks 9 and 12, compared to their baseline levels and to both C groups (Fig. 8b). Reflexive grip strength of TASK-Tx rats did not differ from either C group (Fig. 8b).

Significant differences for “sitting in the corner” (suggestive of task avoidance) were observed for treatment region (*p* = 0.03, Table 1). Post hoc analysis showed that TASK-Ac rats sat in the corner more during the active pulling time period in task week 12, compared to TASK-Tx rats (Fig. 8c). No incidence of fumbling or guarding was observed during task performance across the 12 weeks of task performance by either TASK group.

## Discussion

We have previously observed progressively increasing sensorimotor declines and collagen production in forearm neuromuscular tissues in untreated rats performing this same arduous reaching and grasping task for 3 to 18 weeks [9, 10, 12, 13, 40]. We have reported that a manual therapy protocol based on a combined approach used by many clinical manual therapists (forearm skin rolling, gentle mobilization, upper extremity traction, deeper massage, and wrist joint mobilization) was able to prevent these changes, when provided for 12 weeks concomitant with task performance [12, 13]. In this current study, one goal was to determine if presumed less intense components of the protocol (forearm muscle mobilization and skin rolling, and upper extremity traction and stretch), provided 3 days/week for 12 weeks, concomitant with task performance, also prevented or at least reduced the development of forearm tissue fibrosis and motor declines. We chose these parts of the previously used treatment because our experience indicated that they compress and deform the relevant structures of the rat forearms and forepaws as much as the other treatments, previously presumed to be “deeper” (Fig. 1). We found that the current treatment, similar to our past combined “superficial” and “deeper” treatment, maintained reflexive grip strength in TASK-Tx rats at control levels, and prevented extraneural and muscle fibrogenic changes. The treatment also increased muscle levels of IL-10, and prevented the elevation of CD68+ macrophages in several tissues as well as task-induced tendinopathy, similar to other types of manual therapies, as discussed further below. Additionally, we show for the first time that a manual therapy treatment prevented task-induced elevations of CD206+ macrophages in nerves, muscles, and tendons, as well as cold temperature hypersensitivity.

CD68+ macrophages are pro-inflammatory M1 type macrophages [42, 51, 53]. In the TASK-Ac rats, high numbers of CD68+ macrophages were present in several tissues involved in performing the upper extremity repetitive high force task for 12 weeks, specifically in median nerves, flexor digitorum muscles and tendons, compared to non-task control animals (Fig. 2). IL-1β, a pro-inflammatory cytokine, was also elevated in flexor digitorum muscles of TASK-Ac animals, compared to control animal muscles (Fig. 4a). Pro-inflammatory processes are required for proper muscle repair and clearance of injury-induced debris [54–56]. In this model, CD68+ macrophages and pro-inflammatory cytokines elevate in upper extremity nerves, muscles and tendons either immediately after the initial training period (task week 0) during which rats ramp up from naïve to high force lever pulling, or by task week 3 [7, 10, 12, 40, 52]. The post-training task week 0 is also the peak production point of several pro-inflammatory cytokines (such as, muscle CCL2/MCP-1 and IL-18), while task weeks 3 to 6 are the peaks for others (such as, muscle and serum TNF-α) [7, 10, 19]. Therefore, it was not unexpected that levels of serum TNFalpha, and muscle IL-18 and CCL2/MCP-1 were not elevated at this 12 week TASK time point. However, the continued presence of pro-inflammatory macrophages and muscle IL-1β in 12-week TASK-Ac tissues are indicative of a persistent or chronic task-induced inflammation that may further enhance tissue injuries [54].

Persistent inflammatory responses can also drive an excessive tissue fibrotic repair rather than a restorative repair [30–32, 56], supporting the necessity for preventing or reducing chronic inflammation for the prevention or reduction of tissue fibrosis. We observed high numbers of fibrogenic CD206+ M2a type macrophages in median nerves, flexor digitorum muscles and tendons in 12 week TASK-Ac rats (Fig. 3), and a low but significant increase in muscle IL-10 levels in TASK-Ac rats, compared to non-task control rats (Fig. 4e). Several researchers have reported that CD206+ (mannose receptor+) M2a type macrophages play important roles in fibrosis in lung, liver, skin and muscle [30–32, 57–60]. These report findings are consistent with our observed regional increases in CD206+ macrophages that were paralleled by tissue fibrosis in the same regions, including increased collagen deposition around median nerves (Fig. 5), increased pro-collagen type I deposition and production in endomyseal regions of the flexor digitorum muscle (Fig. 6), and increased tendon cellularity and epitendon cellularity and thickening in mid and distal regions of flexor digitorum tendons (Fig. 7).

In contrast to TASK-Ac rats, the TASK-Tx rats receiving the manual therapy showed no elevation of CD68+ or CD206+ cells in median nerves, flexor digitorum muscles, or flexor digitorum muscles tendons that were still involved in performing the task (Figs. 2 and 3), and no increases in pro-inflammatory cytokines in the flexor digitorum muscles or serum (Fig. 4). A hypothesis that manual/massage therapies can reduce tissue inflammation is supported by similar findings in studies showing reductions of leukocytes and cellular infiltrate in general (assayed using H&E staining) in skeletal muscles after 30 min/day of massage-like cyclic compressive loading for 4 consecutive days that began immediately after a single bout of muscle damaging eccentric exercise in a rabbit model [25, 26]. Similar local cyclic compressive also reduced the numbers of cells expressing TNFα and CCL2/MCP-1 in muscles after hindlimb immobilization [22]. Neutrophil and CD68+ macrophage infiltration also reduce following in vivo self-stretching of the back for 10 min/day, for 2 to 12 days, in a rat model of carrageenan-induced inflammation (carrageenan was injected into subcutaneous tissues of the back) [27, 28]. Massage therapy also reduced serum levels of several pro-inflammatory cytokines (IL-8, TNFα, and CCL2/MCP) to baseline levels in health male athletes following sprint exercise [61].

In this current study, we observed a small but significant increase in IL-10, a key anti-inflammatory cytokine, in TASK-Ac rats, compared to control rats (Fig. 4e), which was not unexpected since CD206+ cells are known to produce IL-10. There was an even larger increase in muscle IL-10 in the TASK-Tx rats, compared to TASK-Ac and control non-task rats (Fig. 4d). This increase in IL-10 may be inhibiting the development of tissue inflammatory responses [62]. IL-10 also appears to have anti-nociceptive effects on muscle pain and pain due to nerve injury [63–65]. Its elevation in the TASK-Tx rats may be contributing to the improved sensorimotor behavioral findings (Fig. 8).

We have previously reported that manual therapy of the upper extremity reduced high intensity task-induced fibrosis in extraneural, muscle, and dermal connective tissues in a long-term study [13], and extraneural fibrosis in a short-term study [12]. We now report that this modified manual therapy similarly reduced task-induced extraneural and muscle fibrosis (Figs. 5 and 6), as well as pathological changes in flexor digitorum endo- and epitendons in TASK-Tx rats despite their continued performance of the high intensity repetitive task (Fig. 7). The increased immune cell scores in TASK-Ac epitendons (Fig. 7l) is explained by the increased CD68+ and CD206+ macrophages in this same tendon region (Figs. 2k and 3k). Other groups have shown similar decreases in inflammation-induced tissue fibrosis after massage/manual therapy. In vivo self-stretching of the back, mentioned earlier in a rat model of carrageenan-induced inflammation, reduced not only inflammation but also the thickness of the lesioned subcutaneous tissue [27, 28]. Manual mobilization or brief active stretching of the back also reduced subcutaneous connective tissue fibrosis induced by subcutaneous microsurgical injury [66]. Furthermore, massage therapy has been shown to increase the numbers of collagen fibrils in tendon regions, suggestive of a beneficial effect of this therapy on tenocyte metabolite activity [67].

Yet, in contrast to collagen type I that were reduced in TASK-Tx muscles, TGF-β1 levels remained high in muscles of both TASK groups, compared to the non task control groups (Fig. 6f). TGF-β1 is known to increase with cyclical loading and mechanical strains on muscles, tendons, and isolated tenocytes or fibroblasts [5, 10, 11, 15, 35, 68, 69]. Our past more complete combination of “superficial” and “deep” manual therapy components lowered TGF-β1 levels in muscles and surrounding connective tissues [13]. In contrast, the current combination of “superficial” components did not. Yet, there was still reduced collagen type I in the TASK-Tx muscles, despite the elevated TGF-β1, supporting past findings that collagen type I production can be independent of TGF-β1 in some contexts. For example, Substance P administration can induce collagen production by tenocytes and lung fibroblasts in vitro and in ex vivo skin explants, independent of TGF-β1 signaling [70–72].

Several task-induced sensorimotor declines were prevented by the manual therapy used in this study. The cold temperature hypersensitivity seen in TASK-Ac rats (Fig. 8a) is a neuropathic pain behavior typically linked to median nerve pathology [11, 73, 74]. In our model, cold temperature hypersensitivity is associated with median nerve inflammation and fibrosis [10, 11, 75], and can be prevented or reduced pharmacologically by blocking signaling of CTGF/CCN2 (connective tissue growth factor/cell communication network factor 2) using a specific monoclonal antibody called FG-3019 or pamreluvmab [11, 40], by blocking substance P signaling using a neurokinin 1 receptor antagonist [75], and here by the early and continued administration of a manual therapy for 12 weeks in TASK-Tx rats. Although the literature is mixed on this topic perhaps due to differences in treatment type, time of administration, assessment tools, and high variability in diagnoses [76, 77], several clinical studies have shown that pain symptoms associated with median neuropathies can improve after massage/manual therapies [76, 78–81]. In further support of the ability of manual therapies to reduce pain behaviors, in vivo self-stretching of the back in the rat model of carrageenan-induced inflammation, reduced mechanical sensitivity as well as the earlier mentioned inflammatory and fibrotic reductions.

Grip strength was maintained at control levels in the TASK-Tx rats (Fig. 8b), similar to our past short- and long-term studies examining the effects of preventive manual therapy on motor behaviors [12, 13]. Reflexive grip strength is a complex neuromuscular behavior that can decline in response to enhanced median compressive neuropathy, nerve or intramuscular inflammatory responses, or muscle fibrosis [14, 35, 48, 49, 52, 82]. Such declines can be prevented with early administration of anti-inflammatory drugs, such as administration of diclofenac sodium within 3 days of exercise-induced muscle damage [83] or the anti-CCN2 drug mentioned earlier [40]. However, neither grip strength declines nor sensory hypersensitivities are restored to control levels when anti-inflammatory drugs are provided during the peak of inflammation or after its decline (such as, with later administration of diclofenac sodium, ibuprofen, or anti-tumor necrosis factor alpha drugs) [9, 18, 19, 83]. These latter findings highlight the need for preventive treatments. Acutely, 4 days of massage like cyclic compressive loading immediately after muscle damage inducing eccentric loading improved muscle viscoelastic properties in anesthetized rats [23, 26, 29], although our reflexive grip strength findings in awake animals are difficult to compare to because inflammation, pain and motivation present in awake animals, are known to effect grip strength in both animals and humans [49, 82, 84].

We are to our knowledge the only laboratory performing true multimodal manual therapy on awake rats, who can provide feedback to the technician treating them. The manual therapy methods that we used were taken directly from clinical practice, only scaled down to the size of the structures. In our experience, trained therapists with no experience with rats readily perform similar treatments without more than a simple description. Therapists continuously adapt their methods using feedback from the patient and the structures. Therefore, the treatment does not need to be described any differently to field practitioners in order to be used.

Some limitations of this study need to be considered. First, only female rats were included. The force transducer sensitivity of our model setup is currently tailored to the pulling strength of female rats, so inclusion of males may have reduced data quality and made the interpretation of findings more difficult, and would have added sex as a potential confounder. Another reason for our focus on one sex is that human females have a higher incidence of work-related musculoskeletal disorders than males [85, 86]. However, human males also develop these disorders [87]. Future studies are encouraged to include males. Additionally, this is an animal study rather than a clinical trial using human patients. That said, a key strength of our model is that it is an operant model in which rats develop changes in the same manner as humans involved in prolonged work-related upper extremity tasks [6].

## Conclusions

These results provide clear evidence that manual therapy provided across several weeks during active performance of an inflammation-inducing task maintains sensorimotor function by lowering nerve, muscle, and tendon inflammation and fibrogenic processes. Combined with our previous reports [12, 13], there is strong support for human clinical trials using similar treatment protocols. Finally, these findings support that the effects of manual therapy are likely local, and not systemic, since the rats receiving similar lower extremity treatment did not benefit in the assays we report.

## Supplementary Information

**Additional file 1: Supplemental Figure 1.** Rat body weights. Animals that performed an operant high intensive reaching and lever bar pulling task for 12 weeks (TASK, *n* = 10 per treatment group) were compared to age-matched control rats (C, *n* = 5 per treatment group). Across these 12 weeks, each group received modeled manual therapy (MMT), bilaterally, to either their upper extremities (treatment, Tx) animals, or to their lower extremities (active control, Ac). A) Rats’ body weights across time, and B) at the time of euthanasia and tissue collection. No significant differences were observed between the four groups across time or at the time of tissue collection. Mean ± 95% CI shown. **Supplemental Figure 2.** Ultrasound detected vocalization results. Groups are as defined in Supplement Figure 1. A) Mean number of calls during treatment. B) Length of calls during treatment. C) Mean frequency (in kHz) of calls during treatment. Mean ± 95% CI shown. **Supplemental Figure 3.** Bonar Scoring results for the proximal region of flexor digitorum tendons. Groups are as defined in Supplement Figure 1. No significant differences were observed between groups. Mean ± 95% CI shown. **Supplemental Figure 4.** Hematoxylin and light eosin stained sections of median nerve branches at the level of the wrist. No neutrophils were visualized in any group’s nerves. The inset in panel A shows an example of a macrophage-like cell.

## Data Availability

The datasets generated during and/or analyzed during the current study are available from the corresponding author (MB) upon reasonable request.

## Abbreviations

°C: Temperature in centrigrade
C-Ac: Non task control rats that received manual therapy treatment to their lower extremities
C-Tx: Non task control rats that received manual therapy treatment to their upper extremities
CCL2/MCP-1: Chemokine (C-C motif) ligand 2/monocyte chemotactic factor 1
CD206: Mannose receptor immunopositive M1 type macrophages known to have fibrogenic roles
CD68: Cluster of differentiation 68, a protein highly expressed by pro-inflammatory cells of the monocyte lineage (phagocytic macrophages) in rodents
cN: centiNewtons
ct: Connective tissue
ELISA: Enzyme linked immunosorbent assay
epi: Epneurium or epitendon (outer connective tissue sheath of nerves or tendons, respectively)
hr: Hour
Hz: Hertz
IL-10: Interleukin 10
IL-1β: Interleukin 1 beta
LE: Lower extremity
M1: Inflammatory type macrophages
M2a: Fibrogenic type macrophages
min: Minutes
mm: Millimeter
MW: Molecular weight
N: Nerve
Pg/ml: Picograms/milliliter
Pg/μg: Picograms/microgram
RAM-II+: An M1 type inflammatory macrophage marker
SEM: Standard error of the mean
TASK-Ac: Rats performing a repetitive high force task that received manual therapy treatment to their uninvolved lower extremities as an active control group
TASK-Tx: Rats performing a repetitive high force task that received manual therapy treatment to task-involved upper extremities
T: Tendon
TGFβ-1: Transforming growth factor beta 1
TNFα: Tumor necrosis factor alpha
UE: Upper extremity
Wk: Week
