# Supplementary material for: Key indicators of repetitive overuse-induced neuromuscular inflammation and fibrosis are prevented by manual therapy in a rat model
Source: BMC Musculoskelet Disord. 2021 May 5;22:417. doi: 10.1186/s12891-021-04270-0 (PMC8101118; doi:10.1186/s12891-021-04270-0)

Supplemental Information

## Key indicators of repetitive overuse-induced neuromuscular inflammation and fibrosis are prevented by manual therapy in a rat model

Mary F Barbe*, Michele Y Harris, Geneva E Cruz, Mamta Amin, Nathan M Billett, Jocelyn T Dorotan, Emily P Day, Seung Y Kim, Geoffrey M Bove

Supplemental Figure 1. Rat body weights. Animals that performed an operant high intensive reaching and lever bar pulling task for 12 weeks (TASK, n=10 per treatment group) were compared to age-matched control rats (C, n=5 per treatment group). Across these 12 weeks, each group received modeled manual therapy (MMT), bilaterally, to either their upper extremities (treatment, Tx) animals, or to their lower extremities (active control, Ac). A) Rats’ body weights across time, and B) at the time of euthanasia and tissue collection. No significant differences were observed between the four groups across time or at the time of tissue collection. Mean ± 95% CI shown.


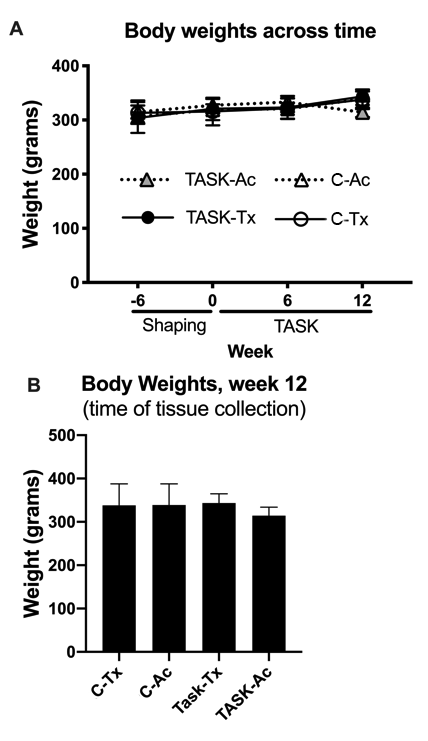


Supplemental Figure 2. Ultrasound detected vocalization results. Groups are as defined in Supplement Figure 1. A) Mean number of calls during treatment. B) Length of calls during treatment. C) Mean frequency (in kHz) of calls during treatment. Mean ± 95% CI shown.


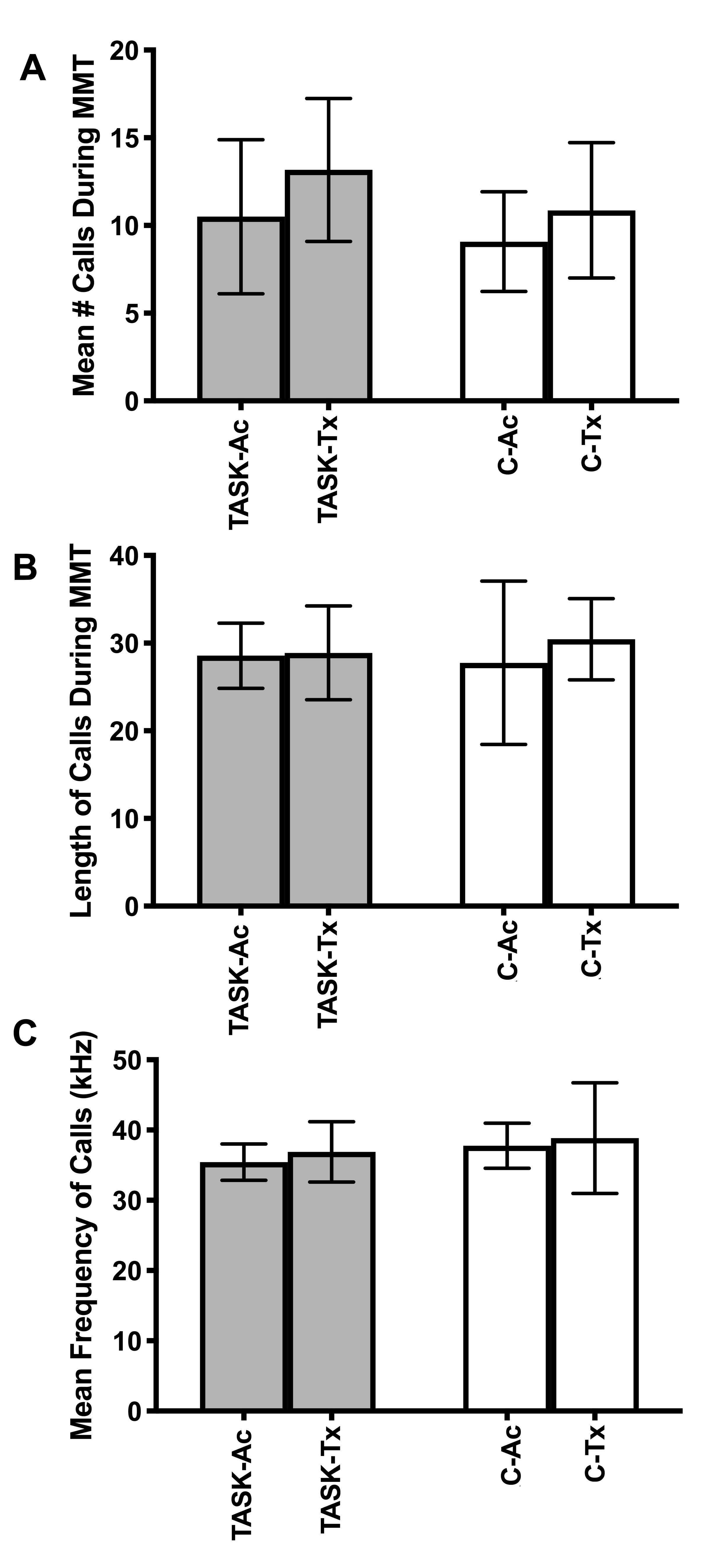


Supplemental Figure 3. Bonar Scoring results for the proximal region of flexor digitorum tendons. Groups are as defined in Supplement Figure 1. No significant differences were observed between groups. Mean ± 95% CI shown.


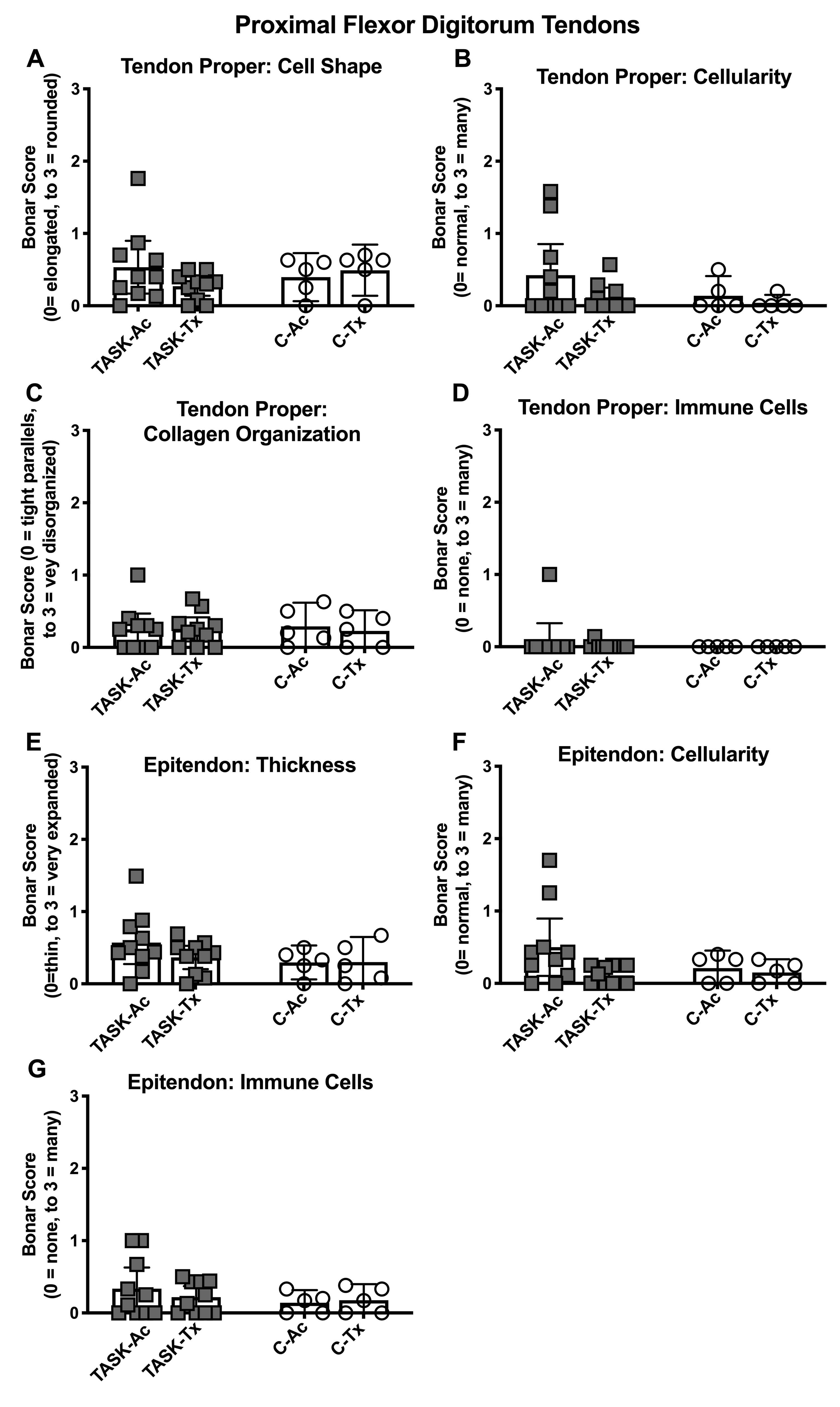


Supplemental Figure 4. Hematoxylin and light eosin stained sections of median nerve branches at the level of the wrist. No neutrophils were visualized in any group’s nerves. The inset in panel A shows an example of a macrophage-like cell.


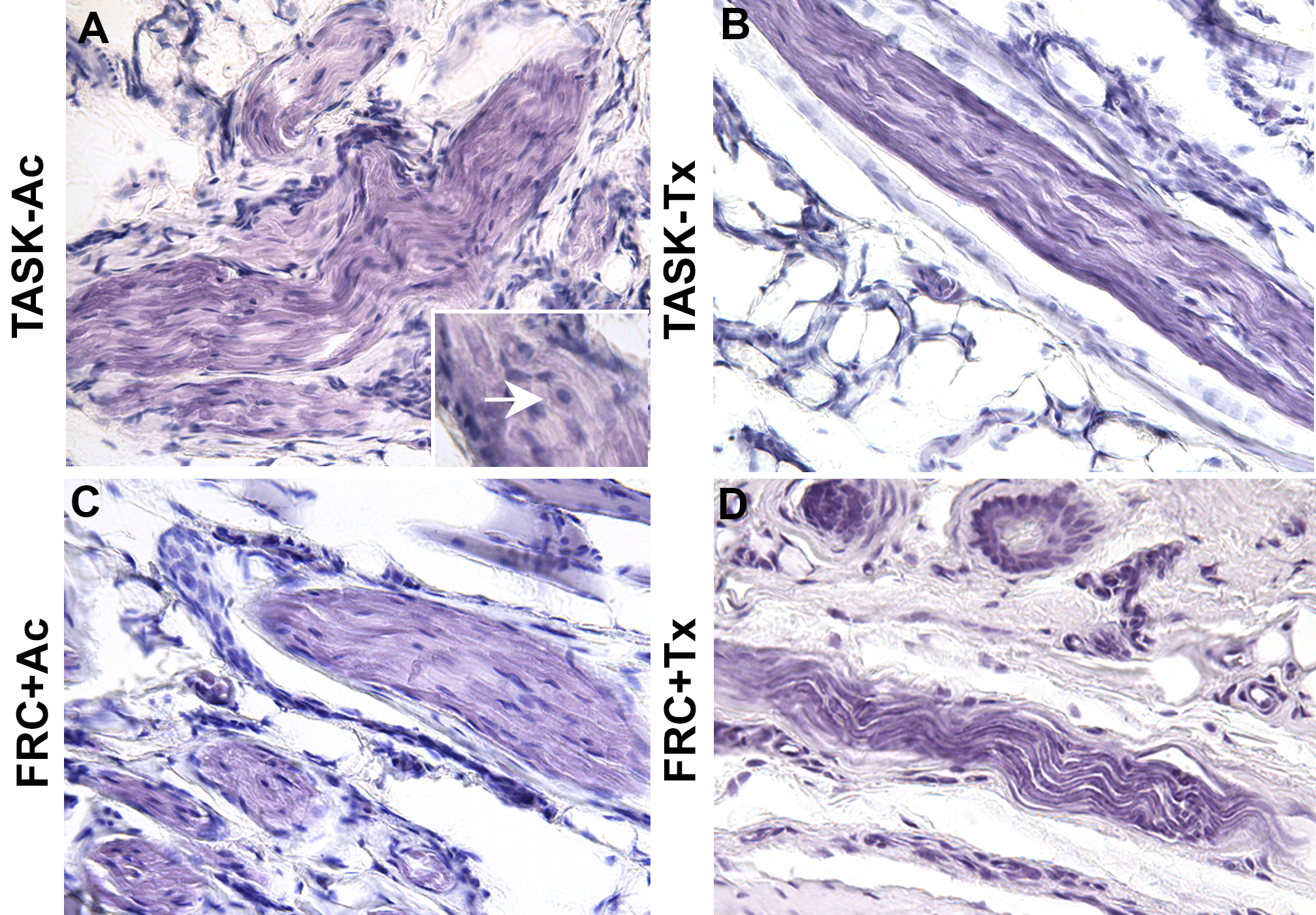

Supplement: Supplementary file 1 — Additional file 1: Supplemental Figure 1. Rat body weights. Animals that performed an operant high intensive reaching and lever bar pulling task for 12 weeks (TASK, n = 10 per treatment group) were compared to age-matched control rats (C, n = 5 per treatment group). Across these 12 weeks, each group received modeled manual therapy (MMT), bilaterally, to either their upper extremities (treatment, Tx) animals, or to their lower extremities (active control, Ac). A) Rats’ body weights across time, and B) at the time of euthanasia and tissue collection. No significant differences were observed between the four groups across time or at the time of tissue collection. Mean ± 95% CI shown. Supplemental Figure 2. Ultrasound detected vocalization results. Groups are as defined in Supplement Figure 1. A) Mean number of calls during treatment. B) Length of calls during treatment. C) Mean frequency (in kHz) of calls during treatment. Mean ± 95% CI shown. Supplemental Figure 3. Bonar Scoring results for the proximal region of flexor digitorum tendons. Groups are as defined in Supplement Figure 1. No significant differences were observed between groups. Mean ± 95% CI shown. Supplemental Figure 4. Hematoxylin and light eosin stained sections of median nerve branches at the level of the wrist. No neutrophils were visualized in any group’s nerves. The inset in panel A shows an example of a macrophage-like cell. [file 12891_2021_4270_MOESM1_ESM.docx]
